# Internal Curing Effect of Pre-Wetted Coal Gangue Powder on Hydration and Shrinkage Behavior of Cement–Fly Ash Composite Materials

**DOI:** 10.3390/ma19204407

**Published:** 2026-10-20

**Authors:** Yonghui Wang, Renzhan Zhou, Chuang Cai, Rongji Zhou, Yuanchao Ou, Dejian Wang, Congcong Wu, Fengxiang Wang

**Affiliations:** 1 College of Civil and Hydraulic Engineering, Bengbu University, Bengbu 233030, Chinawdj0719@163.com (D.W.); ccheu@163.com (C.W.); 19356609718@163.com (F.W.); 2 China Tiesiju Civil Engineering Group Co., Ltd., Hefei 233000, China

**Keywords:** pre-wetting treatment, coal gangue powder, compressive strength, chemical shrinkage, drying shrinkage

## Abstract

**Highlights:**

- Pre-wetted coal gangue powder was used for internal curing of cement–fly ash composites.
- Pre-wetted CGP reduced chemical, autogenous, and drying shrinkage by up to 36.5%, 47.4%, and 36.4%.
- 30% pre-wetted CGP increased 28-day compressive strength by 6.2%.
- Pre-wetted CGP enhanced hydration and refined the pore structure of the composites.
- Pre-wetting offers a simple strategy for improving CGP utilization in cement-based materials.

**Abstract:**

Coal gangue, one of the largest industrial solid wastes generated during coal mining, has accumulated to more than 7 billion tons in China, creating persistent environmental pollution and occupying substantial land resources. Although various activation approaches, including thermal activation, chemical stimulation, and mechanical grinding, have been developed to improve the reactivity of coal gangue, their practical application is still constrained by considerable energy demand and high processing costs. In this work, a simple pre-wetting strategy was introduced to enhance the utilization efficiency of coal gangue powder (CGP) incorporated into cement–fly ash blended binders. Particular attention was given to its influence on shrinkage characteristics and mechanical performance. Pre-wetted CGP replaced the cementitious materials at replacement levels of 10%, 20%, and 30% by mass. Compared with mixtures containing dry CGP, the specimen with 30% pre-wetted CGP exhibited the most pronounced improvement, with chemical shrinkage, autogenous shrinkage, and drying shrinkage decreasing by 36.5%, 47.4%, and 36.4%, respectively, while the 28-day compressive strength increased by 6.2%. These improvements are mainly associated with the internal curing effect provided by the water stored in the pre-wetted CGP, which facilitates cement hydration and results in an 18.6% increase in the intensity of the main hydration peak. In addition, microstructural characterization confirmed that pre-wetting effectively refined the pore system and reduced capillary pore pressure by 33.7%, thereby producing a denser matrix with improved mechanical performance. The proposed pre-wetting treatment offers an effective, economical, and environmentally friendly strategy for the high-value utilization of coal gangue.

## 1. Introduction

The extensive use of coal worldwide has resulted in substantial accumulation of coal gangue (CG), a solid waste constituting 15% to 30% of raw coal production [1]. As the leading coal-producing nation, China currently stockpiles over 8 billion tons of CG, occupying about 160,000 hectares of land, with annual generation surpassing 720 million tons [2]. Although recent utilization rates have reached around 75% [3], conventional approaches remain largely inefficient and economically unviable. CG accumulations pose significant environmental hazards, including risks of spontaneous combustion [4], structural instability, and the release of hazardous elements such as Pb, Cd, and As through weathering mechanisms [5]. These lead to soil pollution, deterioration of air quality, and water contamination, highlighting the critical importance of developing sustainable recycling pathways. In this context, identifying high-volume, low-cost, and environmentally friendly utilization strategies for CG has become an urgent priority in China’s efforts toward sustainable construction and circular economy [6,7]. Importantly, CG consists mainly of SiO_2_ and Al_2_O_3_ (65–75%), resembling the chemical characteristics of pozzolanic substances, which supports its potential application as a supplementary cementitious material (SCM) [8]. The use of CG as an SCM not only offers a viable solution for waste disposal but also contributes to reducing the carbon footprint of cement production, which accounts for approximately 8% of global CO_2_ emissions [9,10].

To effectively utilize CG as an SCM, activation is essential to overcome its inherently low reactivity [11]. Thermal and microwave techniques employ elevated temperatures to decompose kaolinite minerals, thereby enhancing pozzolanic activity [12,13,14]. Chemical activation uses solvents to break down and mobilize silicate and aluminate units from CG, substantially increasing reactivity [15]. Nevertheless, these methods are often hampered by high energy demands and technical complexities, reducing their practical applicability. Mechanical activation, while more energy-efficient [16], tends to produce fine powders with pronounced hygroscopicity and porosity, constraining their broader use. Moreover, the high energy consumption and associated costs of these activation methods have hindered their adoption in large-scale industrial applications, particularly in regions where economic feasibility is a primary concern [17,18]. The incorporation of activated CG in concrete influences mechanical behavior depending on factors such as incorporation ratio and reactivity. For example, Zhao et al. [19] reported that wet-grinding CG for 3 h resulted in a 72% enhancement in compressive strength relative to unprocessed CG. Furthermore, concrete with 10–15% activated CG exhibits superior resistance to freeze–thaw cycles; after 95 cycles, compressive strength reduction was merely 8.8% [20,21]. Nonetheless, widespread adoption is still limited due to energy-intensive processing, dust-related problems, and low substitution thresholds [22], calling for more efficient and scalable activation methodologies. The inherent trade-off between activation efficiency and economic viability suggests that alternative approaches, such as physical modification without intensive energy input, merit further exploration.

In light of the limitations of mechanical activation, pre-wetting has gained attention as a supplementary measure to enhance CG’s efficacy as an SCM. Pre-wetting saturates the internal pores of coal gangue powder (CGP), reducing dust emission and facilitating internal curing. This process diminishes water absorption by recycled aggregates, effectively lowering the water-cement ratio and improving concrete strength, pore structure, and long-term durability [23,24]. Internal curing is a recognized technique in cement-based materials that relies on the controlled release of moisture from saturated porous substrates to mitigate self-desiccation. Compared to conventional external curing methods, internal curing provides a more uniform and sustained moisture supply throughout the cement matrix, which is particularly beneficial for low water-to-binder ratio systems where autogenous shrinkage and early-age cracking are critical concerns [25,26,27]. Yet, pre-wetting applied specifically to CGP has not been thoroughly investigated, especially regarding optimal moisture dosage, kinetics of hydration, and microstructural evolution. Furthermore, the interactions between pre-wetted CGP and other supplementary cementitious materials, such as fly ash, remain largely unexplored, despite the widespread use of binary and ternary binder systems in modern concrete technology. Although studies on other pre-wetted SCM (e.g., copper tailings) reported a 10.2% rise in 28-day strength and 16.2% reduction in autogenous shrinkage [28,29], the distinct physical and chemical properties of CGP demand customized pre-wetting protocols. This research void offers a valuable opportunity to refine the performance of CGP as an SCM while concurrently alleviating environmental and processing issues. Given the abundance and low cost of CG, establishing a simple and effective pre-wetting procedure could significantly promote its practical utilization in construction materials.

Capitalizing on the prospects of pre-wetting treatment, this research comprehensively examines the application of pre-wetted, mechanically activated CGP as a combined replacement for cement and fly ash in blended binder systems. The work assesses the effect of pre-wetting on hydration kinetics and microstructural development, focusing on its capacity to regulate shrinkage behavior. An original contribution of this investigation lies in its dedicated analysis of shrinkage behavior—an essential durability aspect frequently neglected in CG-related studies. Moreover, the combined replacement of both cement and fly ash by CGP, rather than single-component substitution, better reflects the actual formulation of modern blended cements and provides more practical guidance for industrial applications. By tackling both mechanical and durability implications of substituting cement and fly ash with pre-wetted CGP, this study seeks to elucidate the underlying mechanisms of pre-wetting and support the scalable and sustainable use of CG in construction applications. The findings are expected to offer a theoretical basis and technical reference for the value-added utilization of CG, contributing to both environmental protection and resource conservation in the construction sector.

## 2. Experimental Design and Methods

### 2.1. Raw Materials

**Figure 1:**
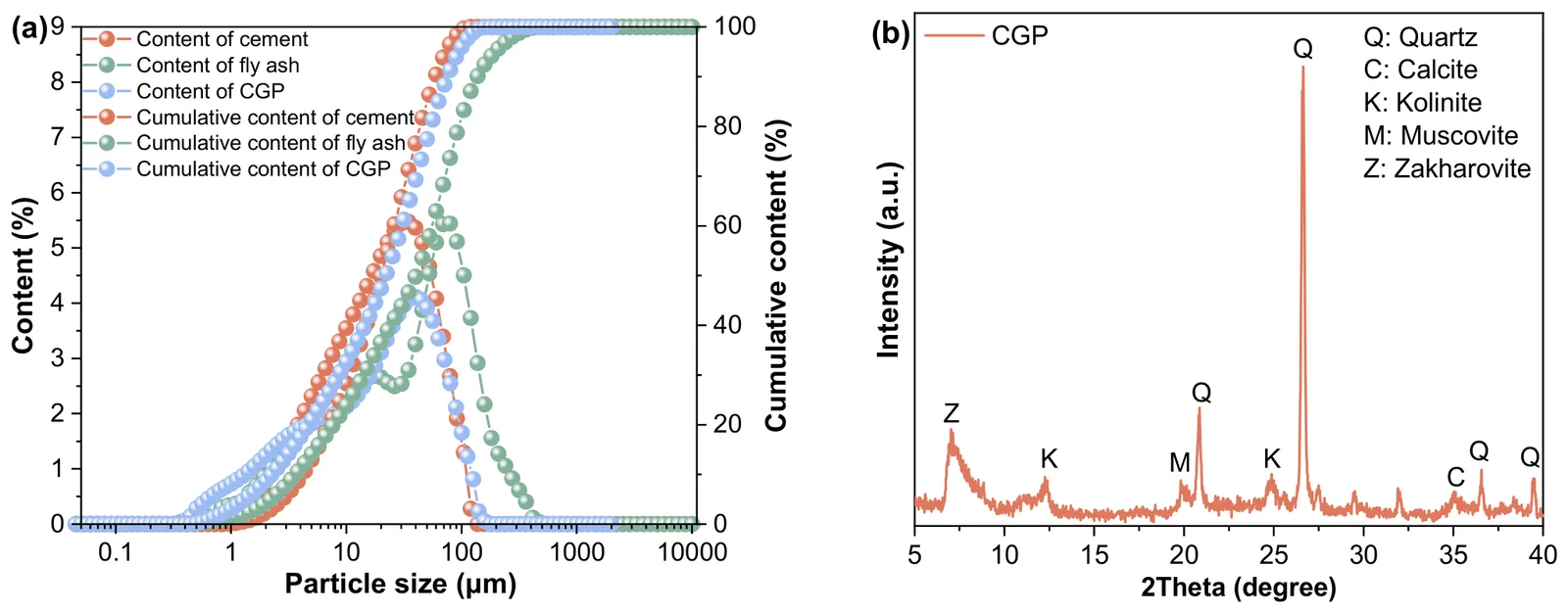
Particle size distributions of cement, fly ash and CGP (**a**) and XRD results of CGP (**b**).

**Figure 2:**
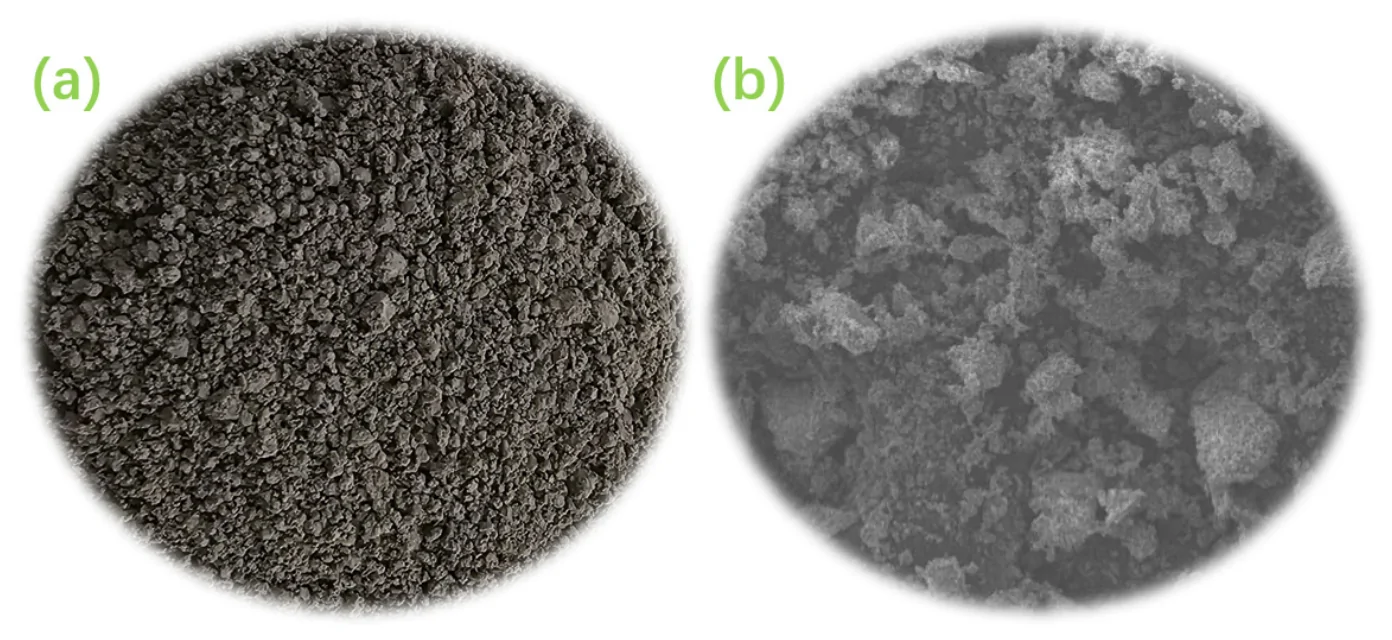
Photograph (**a**) and SEM (**b**) of CGP.

The primary cementitious material used in this study was Type I Portland cement (Anhui Conch Cement Company Limited, Wuhu, China), with measured physical parameters of 3150 kg/m^3^ apparent density and 387 m^2^/kg BET specific surface area. Low-calcium fly ash in its original state as received, without any additional grinding or milling treatment was sourced from a local thermal power plant, showing an apparent density of 2750 kg/m^3^ and a BET surface area of 255 m^2^/kg. CGP, obtained from the Meihuajing Mining Area in Ningxia, China, was mechanically activated by planetary ball milling for 60 min. The apparent density and BET specific surface area of the activated CGP were determined to be 2387 kg/m^3^ and 367 m^2^/kg, respectively. Particle size distribution was characterized by laser diffraction analysis, while the mineralogical composition was identified by X-ray diffraction (XRD), as shown in Figure 1. The main crystalline phases of CGP were calcite, quartz, and kaolinite. The macroscopic appearance and particle morphology of the mechanically activated CGP are shown in Figure 2. Figure 2a presents a photograph of the CGP after planetary ball milling, providing a visual representation of its physical appearance. Figure 2b shows the corresponding SEM image, revealing the irregular particle morphology and rough surface characteristics resulting from mechanical activation. These morphological observations complement the particle size distribution and mineralogical characterization presented in Figure 1 and provide further information on the physical characteristics of the CGP used in this study. In addition, a 24 h water absorption test was conducted, giving a saturated water absorption of approximately 36%. These physicochemical characteristics provide the basis for evaluating the water-storage capacity and physical effects of CGP during the internal curing process.

Table 1 presents the chemical constituents of the raw materials. The CGP exhibits a high SiO_2_ content (57.25 wt.%) but a low CaO content (1.55 wt.%), indicating limited direct contribution to hydration. Although excessive CGP replacement may cause a dilution effect, its pre-wetted state provides internal curing water, which promotes continued hydration and helps maintain compressive strength development, would be analyzed in subsequent chapters.

It should be noted that coal gangue is a heterogeneous solid waste, and its mineralogical composition, particle size, pore structure, and reactivity can vary depending on the coal source and processing history. Therefore, the water absorption capacity and the effectiveness of pre-wetting cannot be assumed to be identical for different CGP sources. The 24 h soaking condition adopted in this study was established based on the measured water absorption behavior of the present mechanically activated CGP, which exhibited an approximate saturated water absorption of 36%. For practical application to other CGP sources, the pre-wetting duration and moisture condition should therefore be calibrated according to their specific water absorption characteristics. In addition, because the CGP investigated in this study showed mainly physical effects with limited direct chemical reactivity, CGP with substantially different mineralogical composition or pozzolanic activity may exhibit different responses to pre-wetting.

### 2.2. Preparation of Samples

The selected CGP replacement levels of 10%, 20%, and 30% were guided by previous studies on coal gangue powder incorporated into cementitious materials [30,31], as shown in Table 2, which investigated replacement levels within a similar range. In the present study, these levels were further adapted to the cement–fly ash composite system to represent low, intermediate, and relatively high CGP incorporation levels. Cement and fly ash were proportionally reduced with increasing CGP content, while the water-to-binder ratio was maintained at 0.33 for all mixtures to ensure a consistent basis for evaluating the effects of CGP incorporation and pre-wetting. For the dry-CGP mixtures (C10, C20, and C30), the 33 g of water was directly added as mixing water. For the pre-wetted-CGP mixtures (CP10, CP20, and CP30), the same 33 g of water was first used to continuously soak the corresponding amount of CGP for 24 h. After soaking, the pre-wetted CGP was not separated from the soaking water; instead, the CGP together with the remaining water was directly added to the other dry constituents for mixing. Therefore, pre-wetting did not introduce additional water into the mixtures or change the total water-to-binder ratio. Rather, it redistributed the fixed amount of mixing water, with part of the water being temporarily stored within the porous CGP particles and subsequently released during hydration. All the paste prepared above was cured in a standard curing room.

### 2.3. Experiments

#### 2.3.1. Setting and Hardening Test

The initial and final setting times of the paste were determined using a Vicat apparatus (Dongfang Huabo Technology Co., Ltd., Beijing, China) in accordance with ASTM C191 [32]. All tests were carried out at a temperature of 20 ± 3 °C and a relative humidity above 95%. Paste flowability was evaluated following ASTM C1437-20 [33] using a flow table (NLD-3, Hangzhou Tongqi Instrument Co., Ltd., Hangzhou, China). The reported value corresponds to the average spread diameter measured along two perpendicular directions. Compressive strength, according to ASTM C192 [34], was measured on 40 mm × 40 mm × 40 mm cube specimens using a universal testing machine (YAW-300D, Jingce Ke Yi Testing Instruments Co., Ltd., Jinan, China). The reported results represent the average of three specimens, and the corresponding standard error was calculated.

#### 2.3.2. Hydration Characteristic

Hydration heat evolution was monitored using an isothermal calorimeter (TAM Air, TA Instruments, New Castle, DE, USA) in accordance with ASTM C1702 [35]. Approximately 50 g of freshly mixed paste was sealed in the testing ampoule immediately after mixing, and the heat flow was continuously recorded for 72 h at a constant temperature of 23 °C. Chemical shrinkage was measured following ASTM C1608 [36]. Three parallel specimens were prepared for each mixture, and the average value together with the standard error was reported. Measurements were initiated 1 h after mixing and continued for 7 days.

#### 2.3.3. Hydration Product

The crystalline phases of the hydrated samples were identified via X-ray diffraction (D8 ADVANCE, Bruker, Karlsruhe, Germany) using Cu-Kα radiation operated at 40 kV and 40 mA. Diffraction patterns were collected over a 2θ range of 5–40° with a scanning rate of 2°/min. Prior to testing, hydration was terminated by immersing the samples in isopropanol for at least 7 days, followed by vacuum drying and grinding to obtain powder passing a 75 μm sieve. Thermogravimetric analysis (TGA) was carried out using a STA 449 F3 thermal analyzer (NETZSCH, Selb, Germany) to quantify the hydration products. Approximately 5 mg of powdered sample was heated from room temperature to 800 °C at a rate of 10 °C/min under a nitrogen atmosphere.

#### 2.3.4. Shrinkage Test

For the internal relative humidity (IRH) test, prepare 100 mm cubic samples and cure them under standard conditions of 23 ± 2 °C and 95% relative humidity. Humidity and temperature measurements were conducted using a digital resistance-based sensor capable (Galltec+Mela, Bondorf, Germany) of simultaneous data acquisition, with accuracies of ±3% for relative humidity and ±0.5 °C for temperature. Sensor readings were automatically recorded through a computerized data acquisition system. Total drying shrinkage was evaluated using standard specimens cast in dismountable iron molds following ASTM C596 [37]. Start recording the initial mass and length after demolding. Subsequently, the samples were stored in an environmental chamber at 23 °C and 50% relative humidity, and the mass loss and dimensional changes were regularly recorded over a period of 28 days. Autogenous shrinkage was determined according to ASTM C1698 [38] using a bellows-based method. Fresh paste was placed in bellows molds, consolidated through vibration, and cured on a wave plate. Following final setting, specimens were transferred to an automated test frame equipped with a displacement sensor (resolution: 1 μm) to monitor linear deformation over a period of 4 weeks. The surface tension of the sample was measured by an automatic interfacial tensiometer (Q2000, Shanghai Fangrui Instrument Co., Ltd., Shanghai, China), with a test range of 0–2000 mN/m and an accuracy of 0.1 mN/m. The measurement was carried out under the condition that the platform lifting speed was 5 mm/min. Each test is completed within 120 s, and the reported value represents the average of three repetitions.

#### 2.3.5. Microstructure

The pore characteristics of the 28-day specimens were analyzed by mercury intrusion porosimetry (AutoPore IV 9500, Micromeritics Instrument Corporation, Norcross, GA, USA). Small fragments collected after the compressive strength test were treated following the hydration-stopping and drying procedure described in Section 2.3.3 before testing. Mercury intrusion was performed up to a maximum pressure of 210 MPa using a surface tension of 0.485 N/m and a contact angle of 130°. The micromechanical properties of the hydrated matrix were evaluated by nanoindentation using a TI Premier Nanoindenter (Hysitron, Minneapolis, MN, USA). Prior to testing, the specimens were vacuum-dried, embedded in epoxy resin, and polished to obtain a smooth surface suitable for indentation [39,40]. Measurements were carried out on a 16 × 16 indentation grid with a spacing of 15 μm. A maximum load of 1 mN was applied during each indentation [41], and the elastic modulus was calculated from the load–displacement curves using the Oliver–Pharr method with a Poisson’s ratio of 0.20 [42].

#### 2.3.6. Use of Generative Artificial Intelligence Tools

ChatGPT-6 (OpenAI, San Francisco, CA, USA) was used to assist in refining the visual presentation of the schematic mechanism diagram, particularly to improve the layout and legibility of the text. The conceptual design, scientific content, mechanistic interpretations, and original draft of the figure were developed and drawn by the authors based on their own research findings and scientific understanding. ChatGPT was used only as an auxiliary tool for visual refinement and did not contribute to the development of the underlying scientific interpretations or the generation of experimental data. The authors reviewed the final figure and take full responsibility for its scientific accuracy and content.

## 3. Results Analysis and Discussions

### 3.1. Performance of Fresh Paste

**Figure 3:**
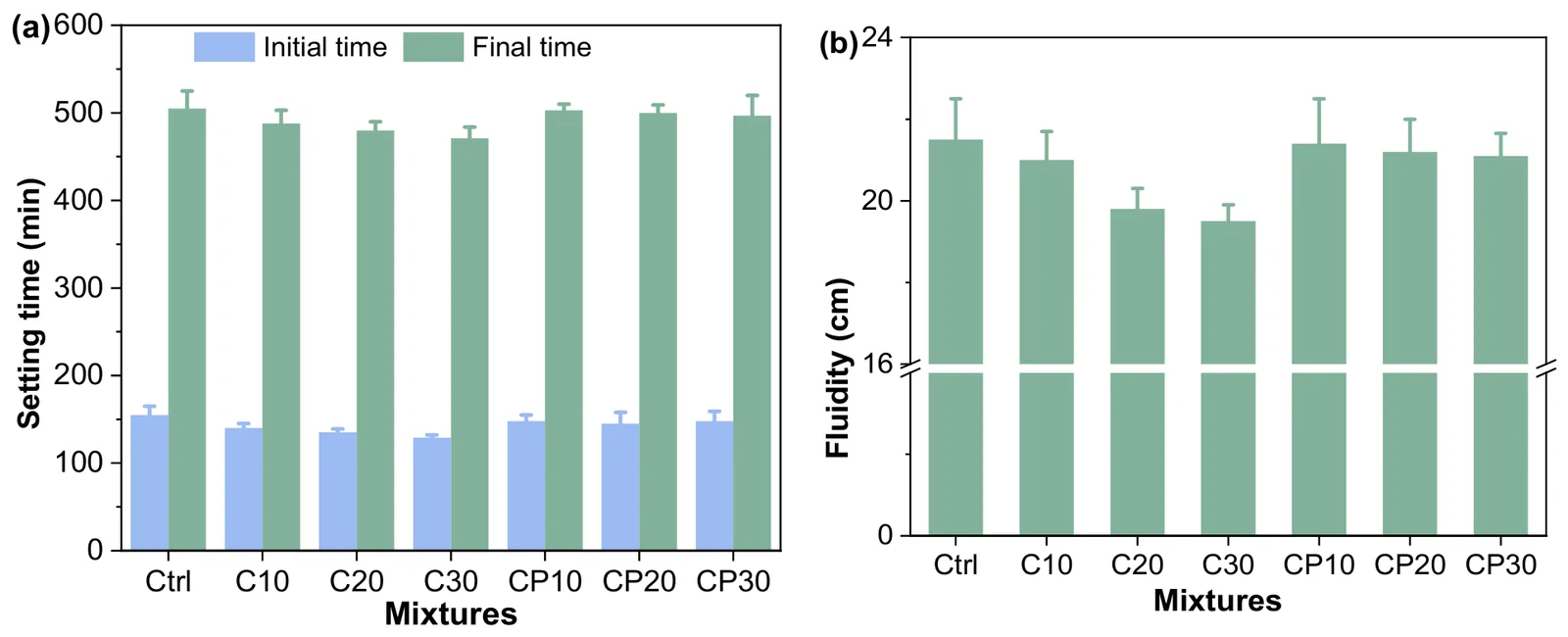
Setting time (**a**) and fluidity (**b**) of samples.

Figure 3a demonstrates the setting characteristics of pastes containing dry and pre-wetted CGP. The addition of dry CGP markedly shortens both initial and final setting times relative to the reference mixture. For example, at a 30% replacement level, the initial setting time decreases to 129 min, while the final setting time is reduced to 471 min. This accelerating effect stems from the pronounced water absorption capacity of dry CGP, which lowers the effective w/b ratio and enhances early hydration kinetics. In comparison, mixtures with pre-wetted CGP display setting behaviors similar to Ctrl, demonstrating that pre-saturation counteracts the water absorption tendency. This stabilization mechanism ensures adequate free water availability, facilitating standard hydration progression and maintaining the properties of the fresh paste.

As depicted in Figure 3b, a comparable pattern is evident in paste fluidity. The use of dry CGP results in a considerable decline in flowability, with the effect intensifying at higher substitution rates. This reduction is caused by the absorption of mixing water by dry CGP particles, diminishing the amount of free water available for lubrication and thereby compromising workability. Conversely, pre-wetted CGP initially improves fluidity at all incorporation levels, attributable to the controlled release of absorbed water during mixing, which enhances particle dispersion and reduces internal friction. As mixing proceeds, the fluidity of pre-wetted CGP mixtures gradually decreases with increasing replacement levels, yet remains consistently higher than that of mixtures containing dry CGP at equivalent dosages. This trend can be explained by the fact that the pre-absorbed water in CGP is gradually released into the paste, compensating for the water consumed by cement hydration and maintaining a more favorable rheological environment [28,29]. Notably, even at the highest replacement level of 30%, the CP30 mixture still exhibits a fluidity of 211 mm, which is comparable to that of the control mixture (215 mm) and significantly higher than that of C30 (195 mm), indicating that pre-wetting effectively mitigates the workability loss typically associated with high-volume CGP incorporation.

### 3.2. Hydration Kinetics

**Figure 4:**
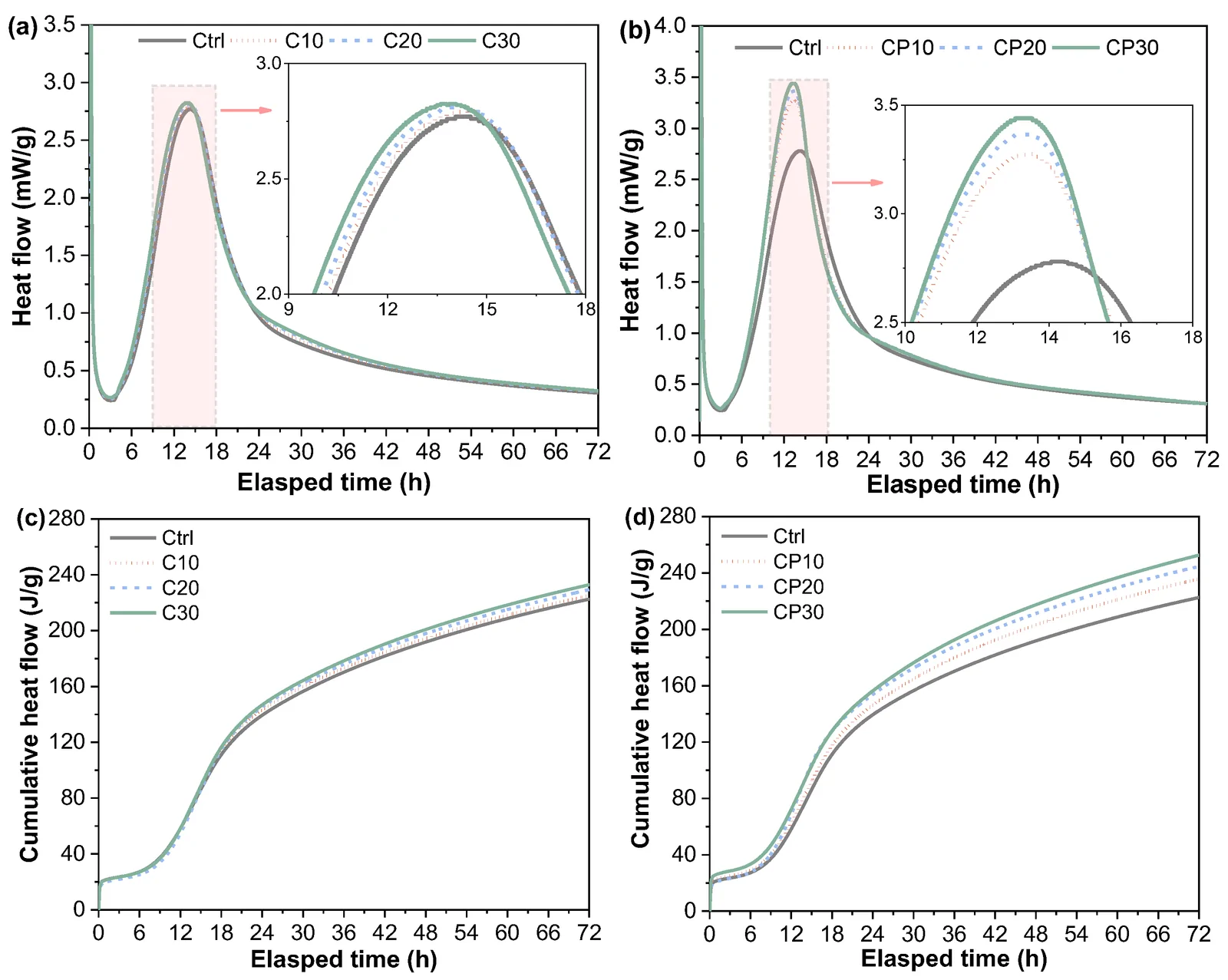
Results of hydration kinetics of pastes: (**a**) heat flow with dry CGP; (**b**) heat flow with pre-wetted CGP; (**c**) cumulative heat with dry CGP; (**d**) cumulative heat with pre-wetted CGP.

Figure 4 presents the hydration heat flow evolution of paste recorded during the initial 72 h. All formulations display two distinct exothermic peaks: the initial peak reflects cement dissolution and early hydration, whereas the second peak corresponds to the main hydration stage characterized by rapid formation of hydration products [43]. The hydration kinetics of cement–fly ash systems are strongly affected by fly ash content and the physical and chemical interactions between cement and supplementary cementitious materials [44]. Isothermal calorimetry has also been widely used to evaluate the effects of supplementary cementitious materials on hydration heat evolution and reaction kinetics [45].

As illustrated in Figure 4a, while CGP incorporation does not alter the fundamental hydration pattern, it substantially intensifies the main exothermic peak. Specifically, mixture C30 demonstrates an 8.8% higher peak heat flow than the control sample. This enhancement may be partly attributed to the nucleation sites provided by the fine CGP particles, which facilitate the precipitation of hydration products. Furthermore, the main peak occurs about 0.5 h earlier in C30 compared with the reference, indicating an accelerated hydration process.

These effects are further amplified when using pre-wetted CGP. At 30% replacement, pre-wetted CGP elevates the main peak intensity by 18.6% and shifts its occurrence forward by 1.2 h relative to Ctrl. This behavior can be reasonably associated with the internal curing effect of pre-wetted CGP. Pre-wetted porous materials can act as internal water reservoirs and release stored water as hydration proceeds, helping to maintain a more favorable moisture condition for continued hydration [46]. The earlier occurrence and higher intensity of the main hydration peak therefore suggest that the combination of fine-particle effects and improved internal water availability contributes to the enhanced hydration kinetics of the pre-wetted CGP mixtures.

The cumulative heat release profiles over 72 h are shown in Figure 4c,d. All mixtures containing CGP generate greater total heat than the control, while the pre-wetted CGP mixtures exhibit a more pronounced increase. This trend is consistent with the combined effects of particle-related physical effects and improved internal water availability. However, the higher cumulative heat release should not be directly interpreted as a quantitative increase in hydration degree because CGP partially replaces the cement–fly ash binder. The calorimetry results are therefore considered together with the TG, internal relative humidity, and microstructural results in the subsequent sections.

The cumulative heat release profiles over 72 h are shown in Figure 4c,d. All mixtures containing CGP generate greater total heat than the control, confirming the material’s contribution to hydration advancement. Specimens with dry CGP show moderate increases in cumulative heat with replacement dosage, consistent with their limited pozzolanic reactivity. For example, C30 exhibits only marginal enhancement compared to the reference. This relatively modest increase suggests that dry CGP primarily acts as a physical filler and nucleation substrate rather than a chemically reactive component during the early hydration period. Conversely, systems incorporating pre-wetted CGP achieve substantially higher cumulative heat release. The value for CP10 already surpasses that of the control, with the difference becoming particularly pronounced in CP30. The progressive increase in cumulative heat with higher pre-wetted CGP content implies that the internal water reservoir effect becomes more effective as more pre-wetted particles are incorporated, ensuring a more complete hydration process over time. These findings unequivocally validate that pre-wetting treatment optimizes both moisture availability and chemical reactivity of CGP, leading to more complete and accelerated hydration processes. Overall, the hydration kinetics results demonstrate that pre-wetting not only enhances the early-age reaction intensity but also sustains the hydration progression over an extended period, which is critical for achieving improved long-term performance of the composite binder system.

### 3.3. Hydration Product of Pastes

#### 3.3.1. XRD Analysis

**Figure 5:**
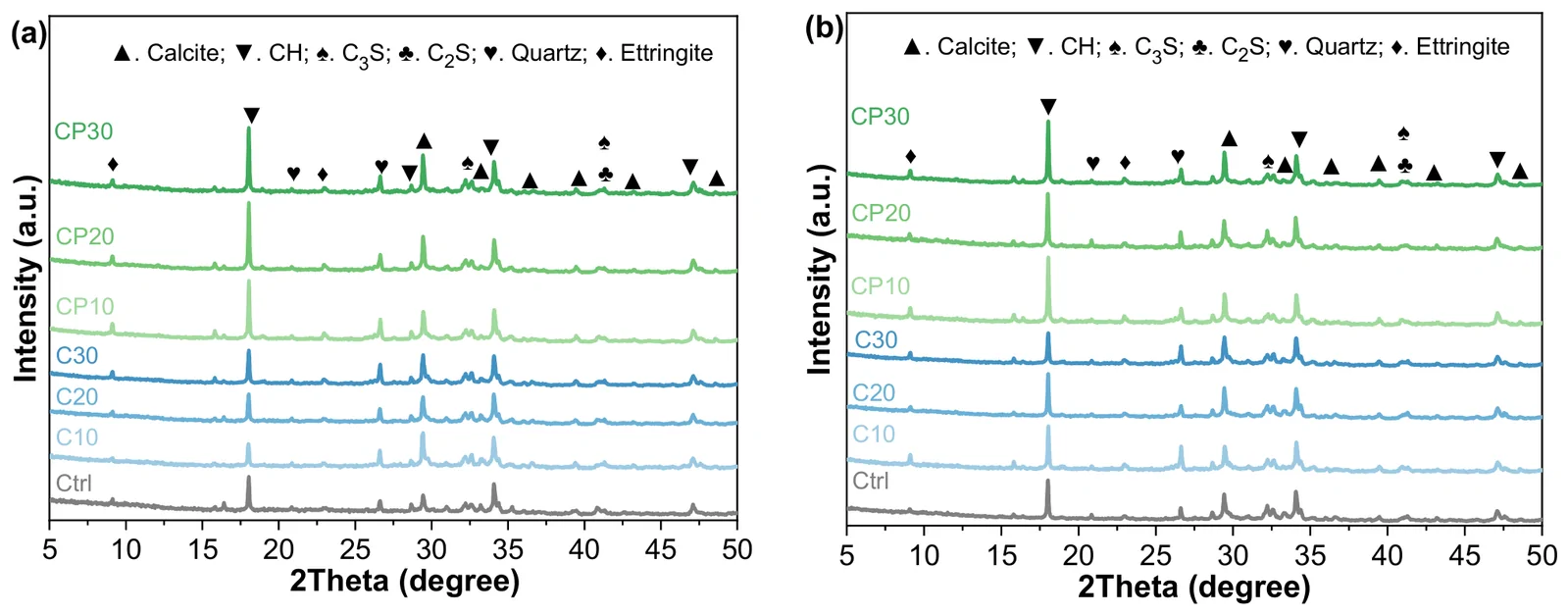
XRD results of pastes: (**a**) 3 days; (**b**) 28 days.

Figure 5 presents the XRD results of paste hydrated for 3 days and 28 days. Major crystalline phases identified include Calcite, Portlandite (CH), Alite (C_3_S), Belite (C_2_S), Quartz, and Ettringite. The phase composition of the hydration products remains largely unchanged, indicating that the influence of CGP is predominantly physical. [11]. It functions as a filler and promotes nucleation without engaging directly in chemical reactions, thereby contributing to the densification of the microstructure. Among these phases, the CH peak near 2θ = 18° is particularly notable. In mixes containing dry CGP, the CH content shows little change, with even a slight reduction in some cases (e.g., C10). This suggests limited pozzolanic consumption of CH and highlights the water-absorbing nature of dry CGP, which may restrict the water available for full hydration. Conversely, the incorporation of pre-wetted CGP led to a significant enhancement in the CH peak. This observed amplification is primarily due to the sustained moisture supply from the internally stored water, which promoted more extensive and prolonged hydration, thereby increasing CH formation. After 28 days of hydration (Figure 5b), the diffraction profiles remain similar, though the CH peak becomes more distinct in mixes with pre-wetted CGP, especially in CP30. These observations collectively validate that pre-wetting optimizes the internal microenvironment, which in turn accelerates hydration kinetics and improves the overall reaction completeness.

#### 3.3.2. TG-DTG

**Figure 6:**
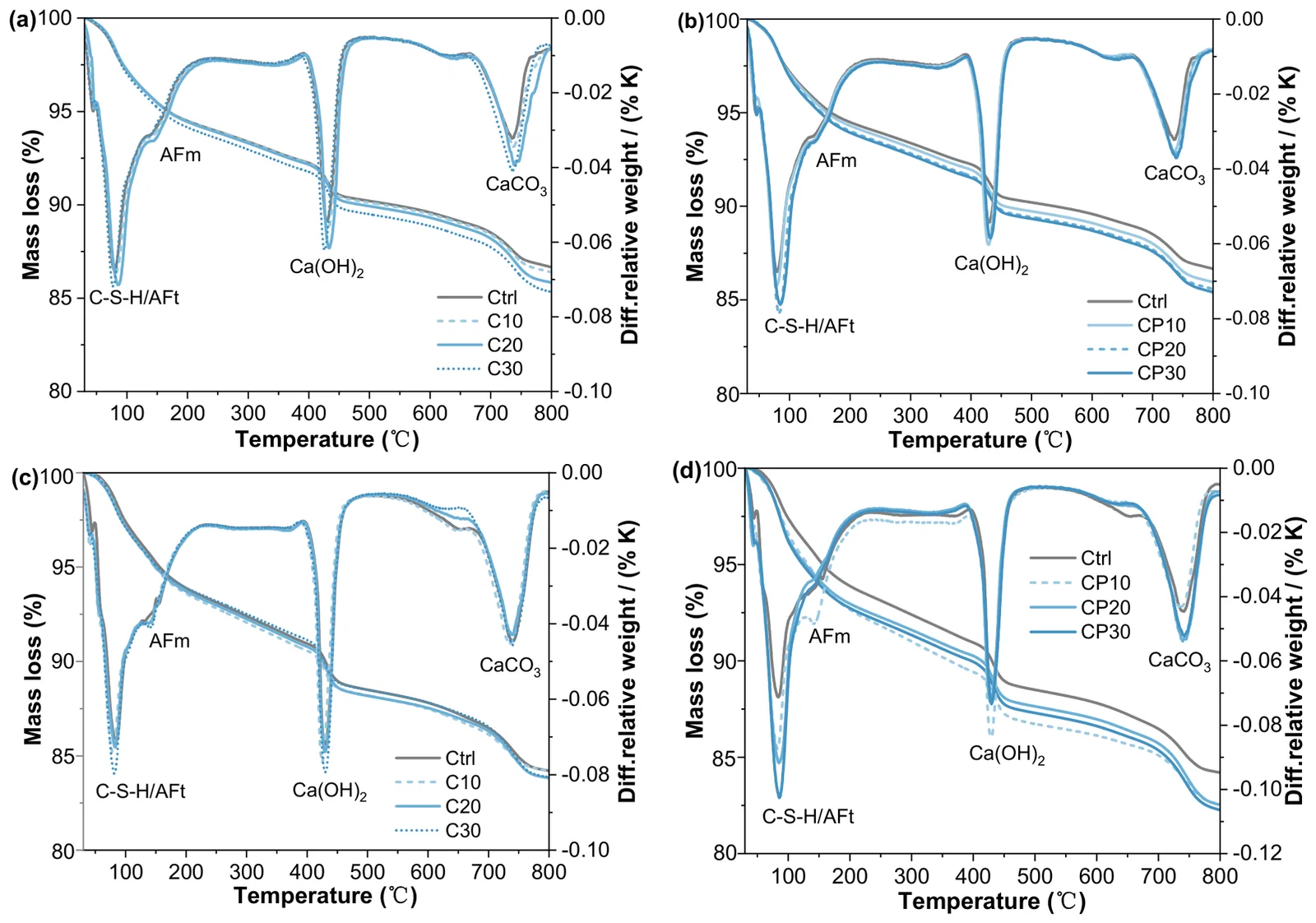
TG-DTG profile of samples: (**a**,**b**) 3 days; (**c**,**d**) 28 days.

Figure 6 depicts the TG-DTG profiles of pastes at 3 days and 28 days, revealing four characteristic endothermic peaks corresponding to the decomposition of C-S-H/AFt (80–105 °C), AFm (180–200 °C), portlandite (400–500 °C), and calcite (650–800 °C) [28,29]. Although all mixes follow a similar decomposition profile, the incorporation of dry CGP leads to a progressive increase in mass loss relative to the Ctrl, indicating enhanced early hydration. This effect is attributed to the fine particle size of CGP, which promotes nucleation sites and may contribute to limited pozzolanic reactivity. Notably, the mass loss increment becomes more evident with higher dry CGP content, suggesting a dose-dependent enhancement of early hydration. The inclusion of pre-wetted CGP leads to a notable rise in mass loss.

Specifically, the CP30 mixture shows a total mass loss of 15.7% after 3 days of curing upon heating to 800 °C, markedly exceeding the values of 14.2% for the control and 14.9% for the dry C30 blend. This elevation is attributed to the internal curing effect, which significantly promotes the generation of hydrates like C-S-H and AFt that decompose upon heating. The retained moisture is gradually released and redistributed via capillary pathways, supporting continued hydration. The greater mass loss observed in pre-wetted CGP mixtures compared to their dry counterparts indicates that the internal moisture supply not only enhances the total quantity of hydration products but also promotes a more uniform distribution of these products within the paste matrix. By 28 days, the mass loss values converge across mixtures, though samples with higher CGP content still show slightly elevated losses. Notably, pre-wetted CGP continues to demonstrate a clear enhancing effect, with CP30 reaching 19.1% mass loss. The convergence trend at later ages suggests that while pre-wetting accelerates early hydration, the overall hydration degree at 28 days is primarily governed by the total binder composition, with CGP acting as a supplementary component that marginally increases the total hydrate content. These findings underscore the role of pre-wetting in sustaining hydration over time through improved internal moisture supply.

### 3.4. Shrinkage Analysis

#### 3.4.1. Chemical Shrinkage

**Figure 7:**
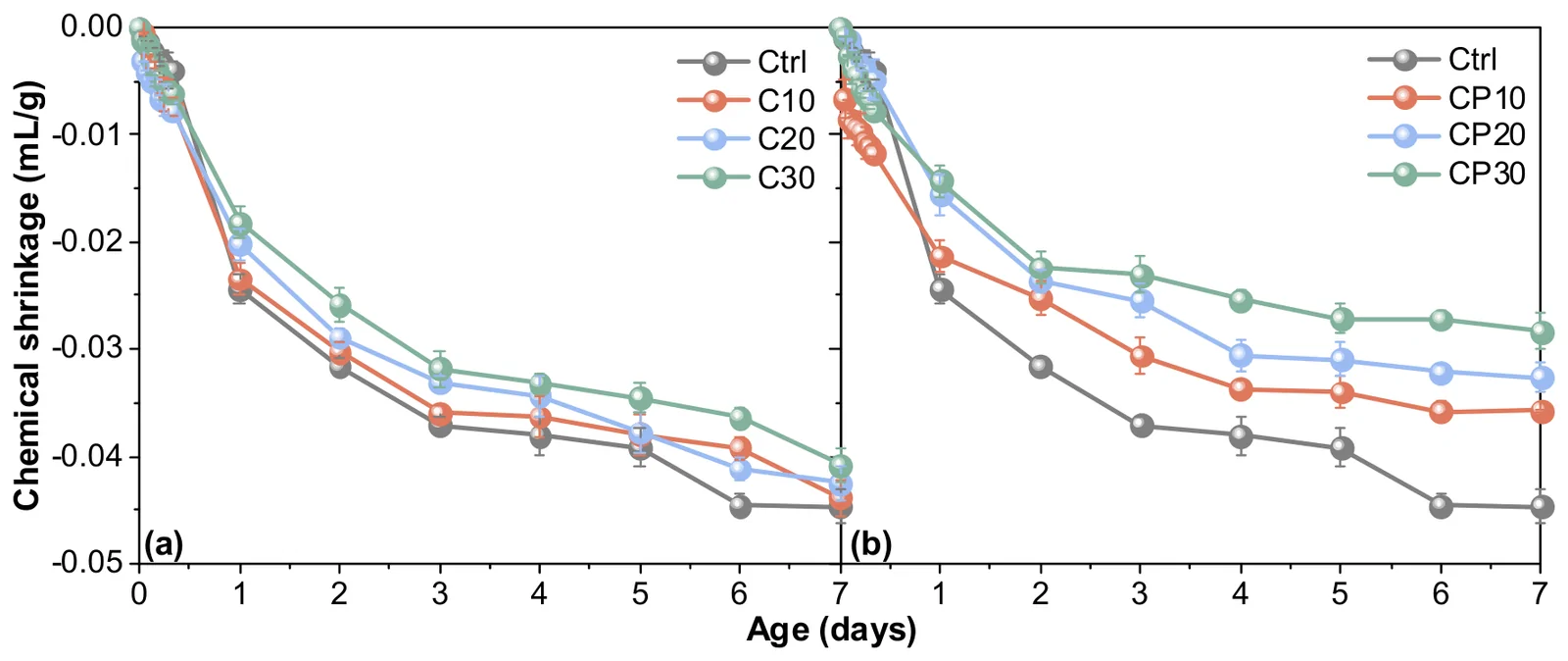
Chemical shrinkage of samples over 7 days. (**a**) conventional mixtures; (**b**) pre-wetted mixtures.

The evolution of chemical shrinkage for all mixtures during the first 7 days is illustrated in Figure 7. Regardless of the mixture composition, chemical shrinkage continuously increased with curing age, reflecting the progressive consumption of water associated with cement hydration. For mixtures containing dry CGP, replacing cementitious materials with 10%, 20%, and 30% CGP resulted in a slight reduction in the 7-day chemical shrinkage, corresponding to decreases of approximately 1.7–8.6% compared with the control specimen. This behavior is mainly attributed to the micro-filler and nucleation effects of the finely ground CGP particles, which provide favorable sites for the precipitation of hydration products and promote the early hydration reaction, thereby only slightly influencing the overall chemical shrinkage [47].

A markedly different trend was observed after introducing pre-wetted CGP. Although chemical shrinkage increased rapidly during the initial hydration period, its growth rate gradually decreased after approximately 3 days. Moreover, the reduction became more pronounced with increasing pre-wetted CGP content, indicating that the internal curing effect was progressively enhanced. At replacement levels of 10%, 20%, and 30%, the 7-day chemical shrinkage was reduced by 20.0%, 27.1%, and 36.5%, respectively, relative to the control mixture. A markedly different trend was observed after introducing pre-wetted CGP. Although chemical shrinkage increased rapidly during the initial hydration period, its growth rate gradually decreased after approximately 3 days. At replacement levels of 10%, 20%, and 30%, the 7-day measured chemical shrinkage was reduced by 20.0%, 27.1%, and 36.5%, respectively, relative to the control mixture. This reduction should not be interpreted as a decrease in hydration. Instead, pre-wetted CGP redistributed the fixed mixing water within the paste and provided an internal water reservoir that was gradually released during hydration. The sustained internal moisture supply mitigated self-desiccation and maintained a more favorable moisture condition for continued hydration. Consequently, the measured chemical shrinkage of the composite system was reduced while hydration proceeded more continuously. This additional internal water supply effectively alleviates self-desiccation and maintains a higher internal moisture condition, thereby facilitating hydration during the early stage [48]. Consequently, hydration products are generated more uniformly and gradually fill the pore space, resulting in a denser microstructure. As hydration proceeds, the increasingly compact matrix reduces the rate of subsequent hydration and pore evolution, leading to a progressive decline in the growth rate of chemical shrinkage after the first few days. Therefore, mixtures containing pre-wetted CGP exhibit a rapid increase in chemical shrinkage at early ages, followed by a substantially moderated development during the later curing period.

#### 3.4.2. Internal Relative Humidity (IRH)

**Figure 8:**
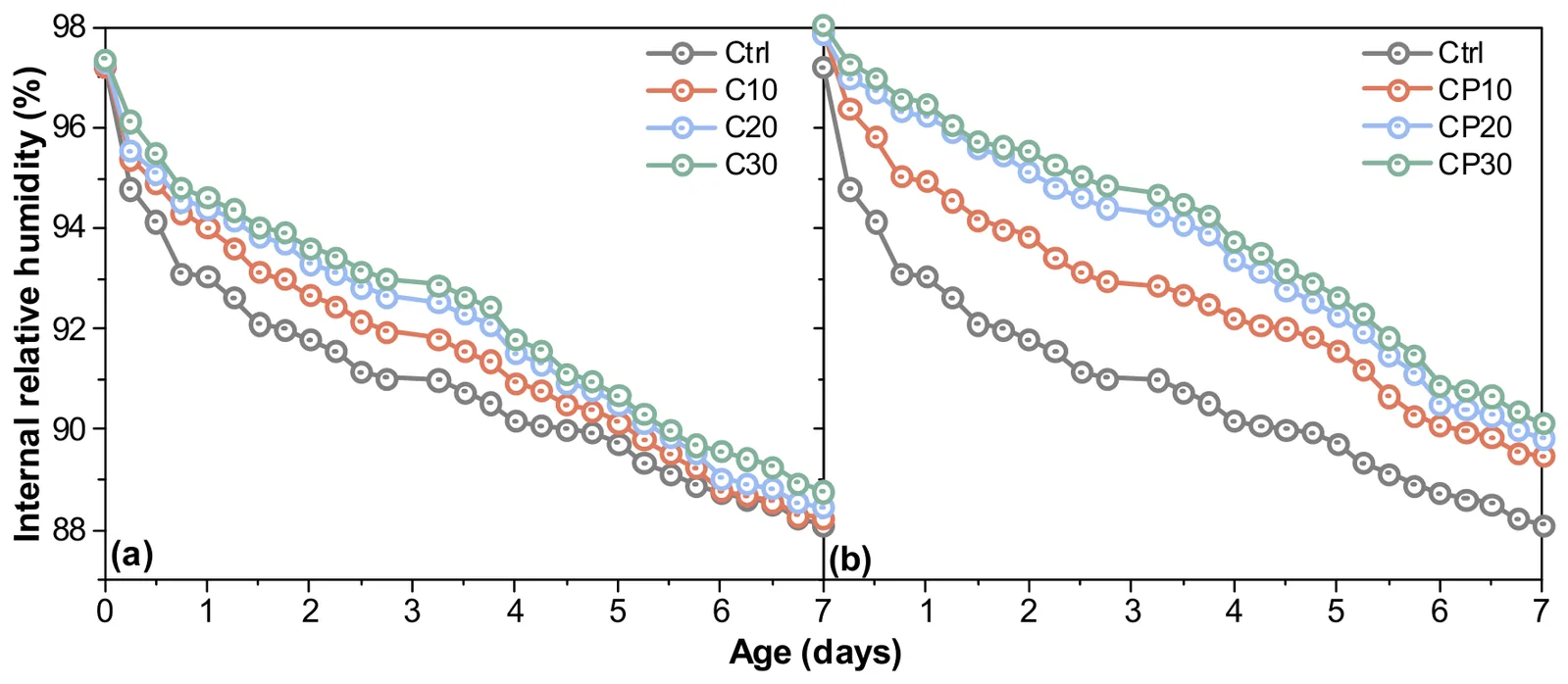
IRH result of paste over 7 days. (**a**) conventional mixtures; (**b**) pre-wetted mixtures.

The variation in IRH for all mixtures during the 28-day curing period is presented in Figure 8. A continuous decline in IRH was observed for all specimens with increasing curing age, which is mainly related to the progressive consumption of internal water during cement hydration and the development of capillary self-desiccation. For the control mixture, IRH decreased from 97.2% to 88.1% within the first 7 days, indicating a significant reduction in internal moisture availability caused by hydration reactions. Compared with the control sample, the incorporation of dry CGP slightly increased the IRH values. Although all dry CGP-containing mixtures exhibited a similar downward trend, the C30 mixture maintained a relatively higher humidity level throughout the curing process. This phenomenon can be explained by the water regulation capability of CGP particles. During mixing, dry CGP initially absorbs part of the available water, while the absorbed moisture can be gradually released during subsequent hydration, providing a limited internal curing effect and mitigating moisture loss within the cement matrix.

A more significant improvement in IRH was achieved when pre-wetted CGP was incorporated. Compared with dry CGP, the pre-saturated particles acted as internal water reservoirs and continuously supplied additional moisture during hydration, effectively compensating for water depletion and maintaining a higher internal humidity environment. Furthermore, the beneficial effect became increasingly evident with increasing pre-wetted CGP content. At 7 days, the IRH of the CP30 mixture reached 90.1%, which was 2.0% higher than that of the control and 1.3% higher than that of the corresponding dry CGP mixture (C30). The enhanced moisture retention provided by pre-wetted CGP facilitates sustained hydration and promotes the gradual formation of hydration products, contributing to pore refinement and matrix densification. Therefore, the incorporation of pre-wetted CGP effectively regulates internal moisture conditions and provides a favorable environment for long-term hydration development.

#### 3.4.3. Dry Shrinkage Analysis and Mass Loss

**Figure 9:**
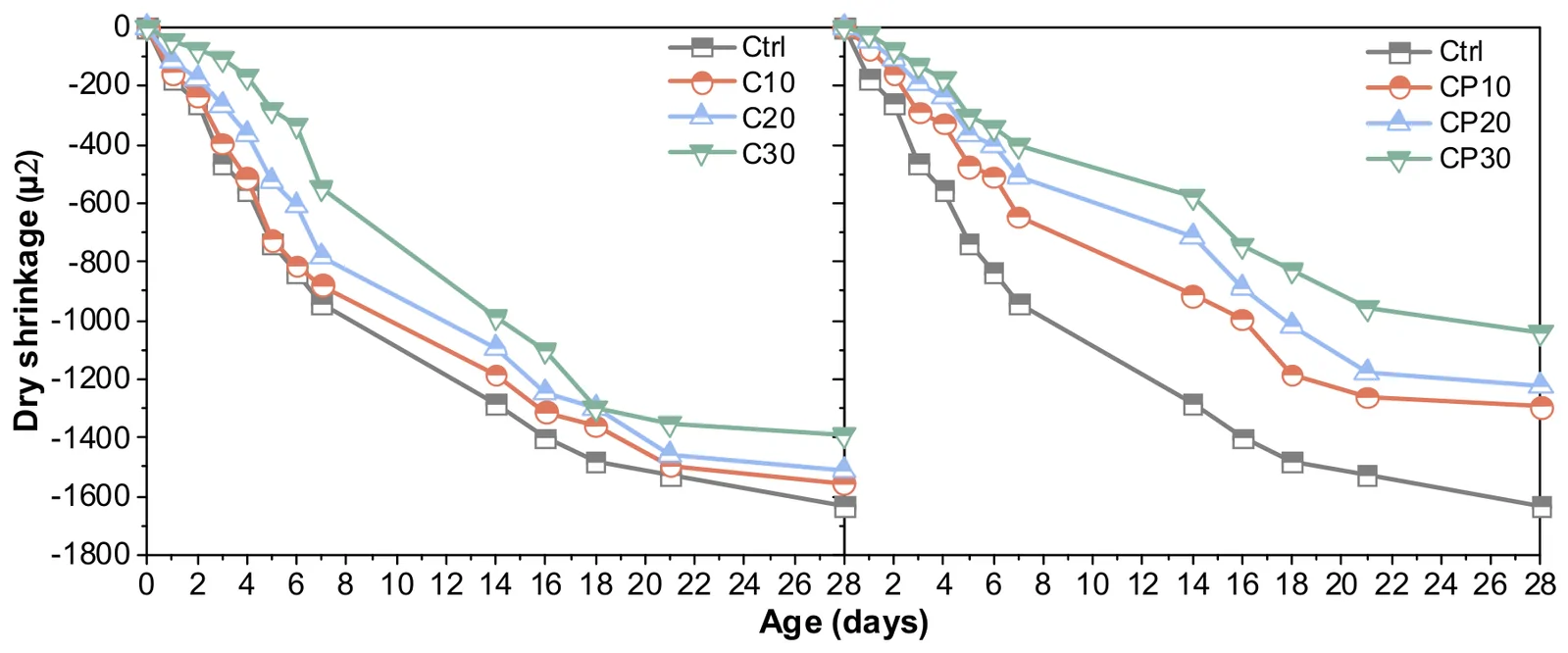
Drying shrinkage results for all mixtures at 28 days.

Figure 9 displays the 28-day drying shrinkage behavior of mixtures incorporating dry and pre-wetted CGP. Drying shrinkage develops due to moisture evaporation from capillary pores under low-humidity conditions [49]. The inclusion of CGP modifies the pore connectivity, facilitating the escape of free water, yet simultaneously contributes to a reduction in overall shrinkage. With the replacement level increases, drying shrinkage declines consistently, with the most significant reduction observed at 30% substitution. This mitigating effect is attributed to the enhanced degree of hydration promoted by CGP, which refines the pore structure and increases resistance to deformation. The use of pre-wetted CGP leads to further suppression of shrinkage. A clear divergence in shrinkage behavior emerges from day 5 onward between mixtures containing pre-wetted CGP and Ctrl, continuing throughout the testing period. At 28 days, the incorporation of pre-wetted CGP at 10%, 20%, and 30% replacement levels led to drying shrinkage reductions of 20.6%, 25.1%, and 36.4%, respectively. This suppression of shrinkage is attributed to the refined pore structure and improved hydration product morphology resulting from internal curing, which collectively enhance the matrix’s resistance to moisture loss and improve its long-term dimensional stability.

**Figure 10:**
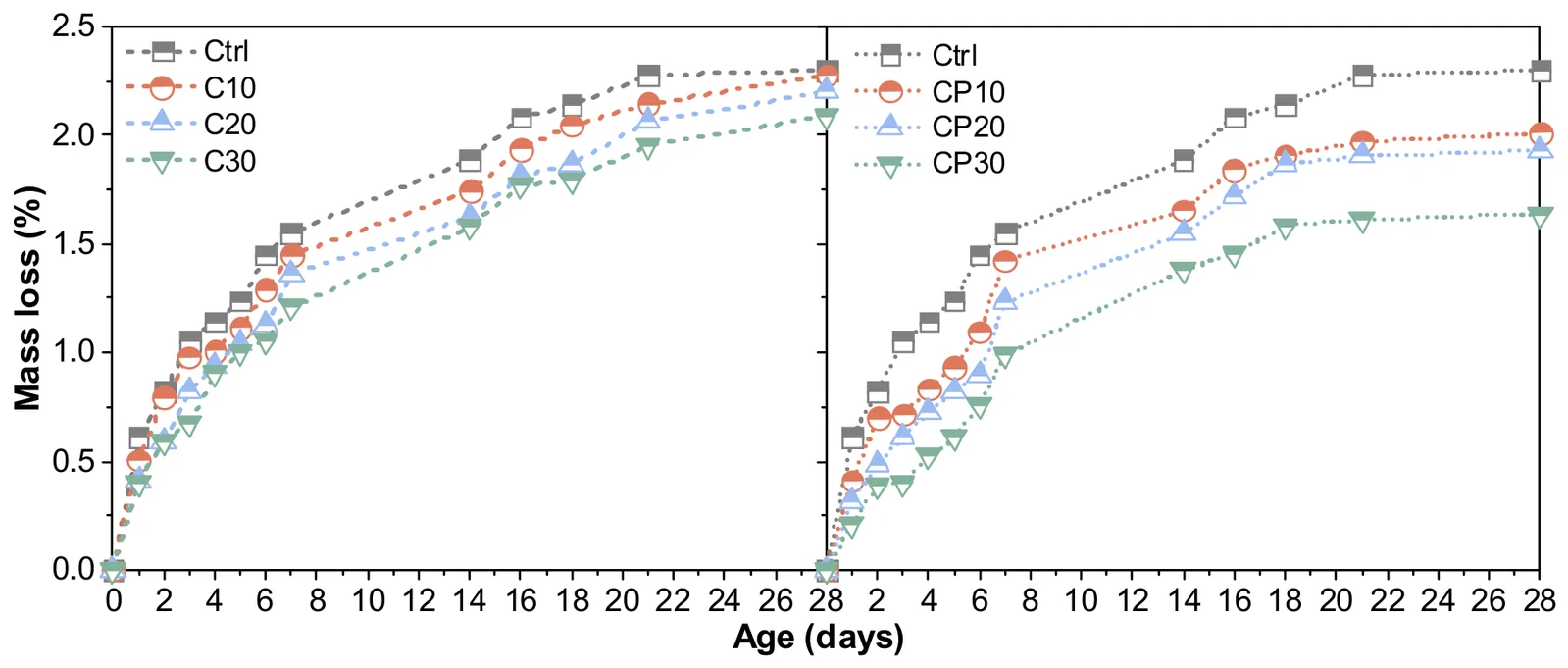
Mass loss results for all mixtures at 28 days.

The mass loss evolution of the CGP-containing pastes after 28 days of curing is illustrated in Figure 10. All mixtures exhibited a continuous decrease in mass during the drying process, with more than 55% of the total mass reduction occurring within the first 7 days. This indicates that early-age moisture migration plays a dominant role in determining the subsequent drying behavior of the cementitious system. Compared with the control mixture, the incorporation of dry CGP slightly reduced the rate of mass loss. This phenomenon can be associated with the water regulation capability of CGP particles, which partially retain internal moisture and contribute to the hydration process, thereby reducing the amount of free water available for evaporation. A more evident reduction in mass loss was observed for mixtures containing pre-wetted CGP. At identical replacement levels, the pre-wetted CGP mixtures consistently exhibited lower mass loss than their corresponding dry CGP counterparts. Although the pre-wetting treatment introduced additional water into the system, the internally stored moisture was gradually released during hydration, promoting a higher degree of reaction and the formation of additional hydration products. These newly generated products contribute to pore refinement and matrix densification, which reduce the connectivity of evaporation pathways and suppress subsequent moisture loss. Therefore, pre-wetted CGP demonstrates a more effective capability in regulating water migration and improving the drying resistance of cement-based composites.

#### 3.4.4. Autogenous Shrinkage

**Figure 11:**
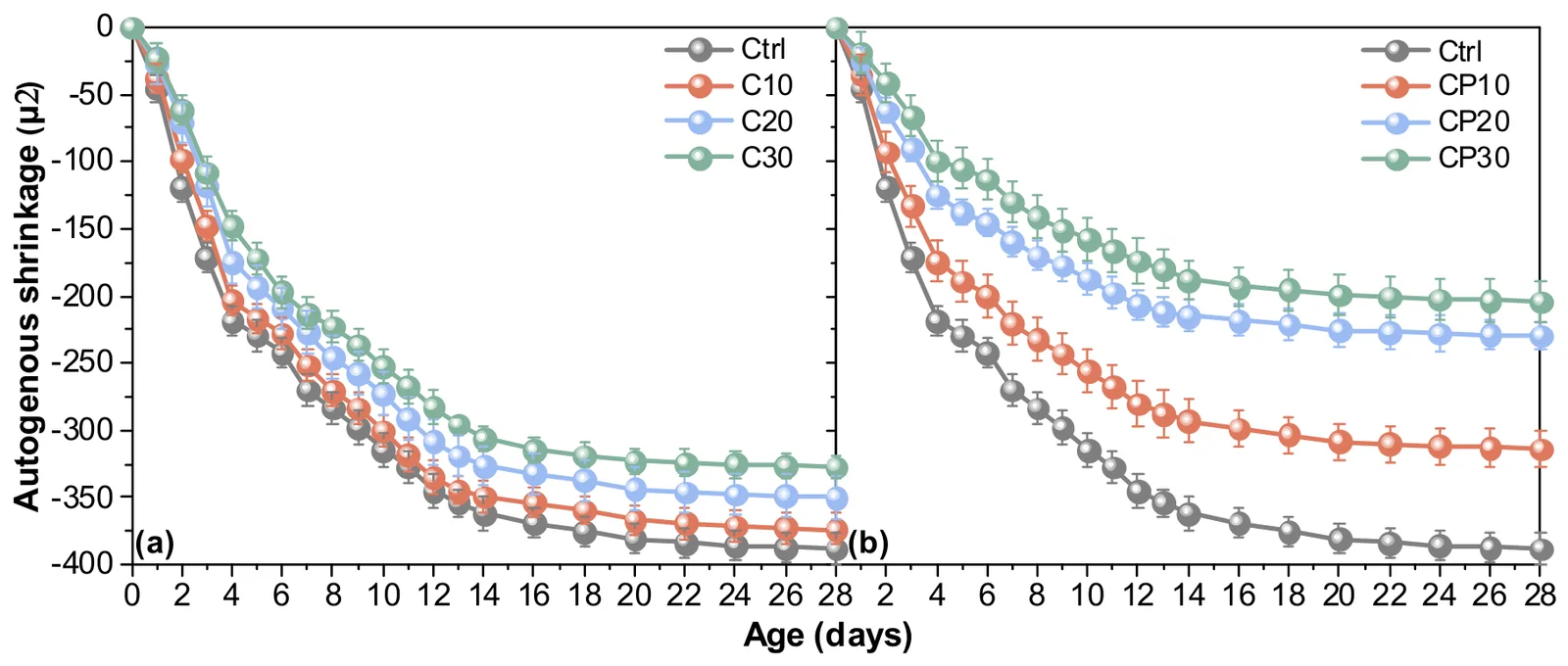
Autogenous shrinkage results for all mixtures at 28 days. (**a**) conventional mixtures; (**b**) pre-wetted mixtures.

Figure 11 illustrates the development of autogenous shrinkage in cement pastes over a 28-day period. The control sample exhibits significant autogenous shrinkage, reaching 361 µε by day 14 and stabilizing at 388 µε at 28 days, indicating a heightened risk of early-age cracking. This pronounced shrinkage in the control mixture is primarily attributed to self-desiccation caused by ongoing cement hydration in a sealed system with no external water supply. The incorporation of dry CGP leads to a moderate reduction in autogenous shrinkage, with values decreasing to 373 µε (C10), 349 µε (C20), and 327 µε (C30), corresponding to reductions of 3.9%, 10.1%, and 15.7% compared to the control. This reduction results from the gradual release of moisture previously absorbed by dry CGP into the capillary system, which partly offsets chemical shrinkage.

However, the effectiveness of dry CGP in mitigating autogenous shrinkage is limited, as the absorbed water may not be entirely available for internal curing due to the strong capillary forces within the fine pores. Pre-wetted CGP exhibits a more significant effect, reducing autogenous shrinkage to 313 µε (CP10), 229 µε (CP20), and 204 µε (CP30)—corresponding to substantial decreases of 19.3%, 41.0%, and 47.4% compared to the control. The marked difference between dry and pre-wetted CGP mixtures highlights the critical role of pre-saturation in ensuring that the stored water is readily available for timely release during the hydration process. In contrast to dry CGP’s initial water absorption, the pre-saturated particles function as an internal reservoir, providing continuous moisture supply throughout the hydration process. This mechanism maintains higher internal humidity levels, significantly reducing self-desiccation and associated shrinkage, thereby effectively enhancing crack resistance [50]. The superior performance of pre-wetted CGP in suppressing autogenous shrinkage is consistent with the IRH results presented in Section 3.6, where CP30 exhibited the highest internal relative humidity throughout the testing period, confirming that sustained moisture availability is the key factor in reducing autogenous deformation.

### 3.5. Compressive Strength

**Figure 12:**
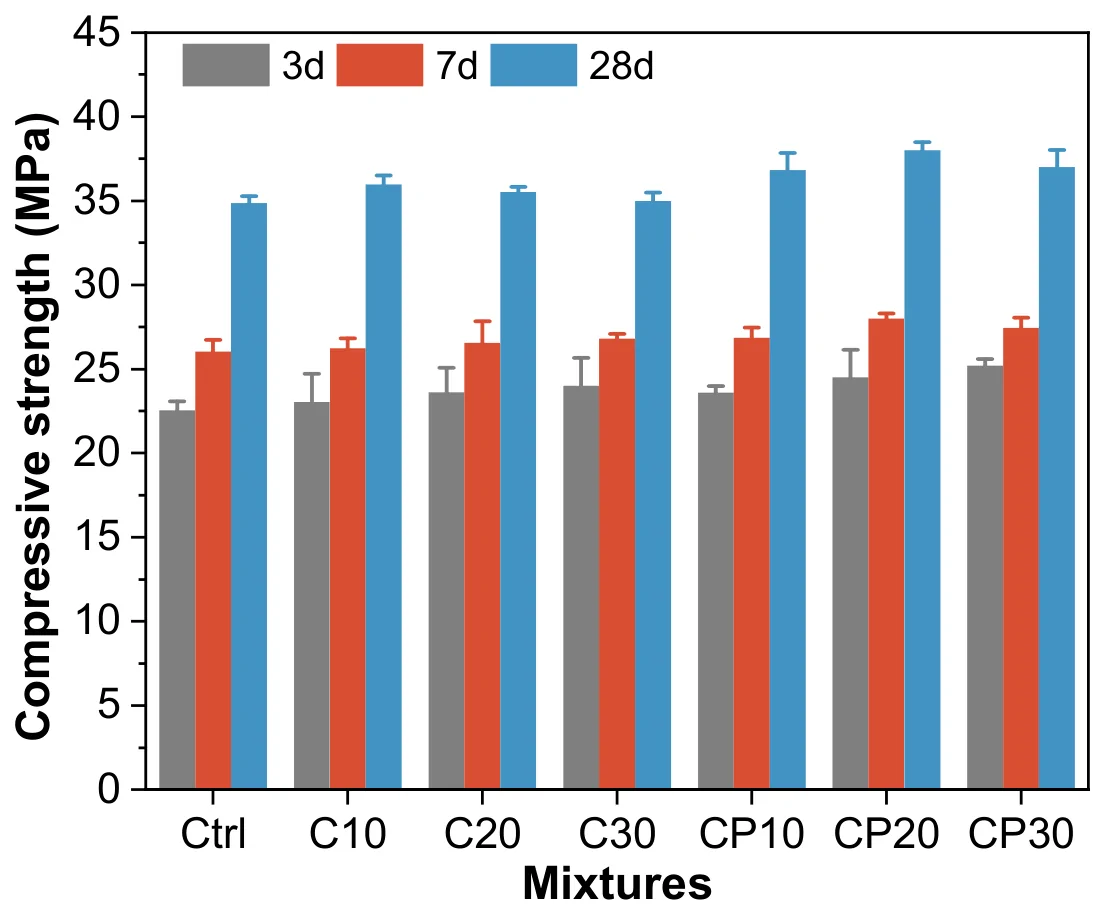
Compressive strength of pastes.

Figure 12 shows the development of compressive strength in cement pastes with different CGP treatments over time. During the early stages, mixtures with dry CGP display a consistent strength gain. The C30 formulation, for instance, achieves a 6.5% higher strength than the control at 3 days, maintaining a 2.9% advantage by 7 days. This early-age improvement is linked to the internal curing capability of dry CGP, whereby the gradual release of stored moisture facilitates prolonged hydration and promotes the formation of strength-bearing phases like C-S-H gel. However, after 28 days, the compressive strength tends to decrease with higher dry CGP content. Although C30 still shows slightly higher strength than the control, the overall reduction suggests that the porous and weakly bonded nature of dry CGP may introduce microstructural defects over time, facilitating crack initiation and limiting long-term performance [51]. In contrast, pastes incorporating pre-wetted CGP demonstrate consistent strength gains across all ages. CP30, for instance, achieves strength increases of 11.7%, 5.5%, and 6.2% at 3, 7, and 28 days, respectively. The pre-wetted CGP acts as an efficient internal water reservoir, ensuring sustained moisture supply for hydration and promoting microstructural refinement through the continued precipitation of hydrates. The most favorable performance is observed at 20% replacement, where an optimal combination of enhanced hydration and pore structure densification yields the highest 28-day strength.

### 3.6. Microstructure Analysis

#### 3.6.1. MIP

**Figure 13:**
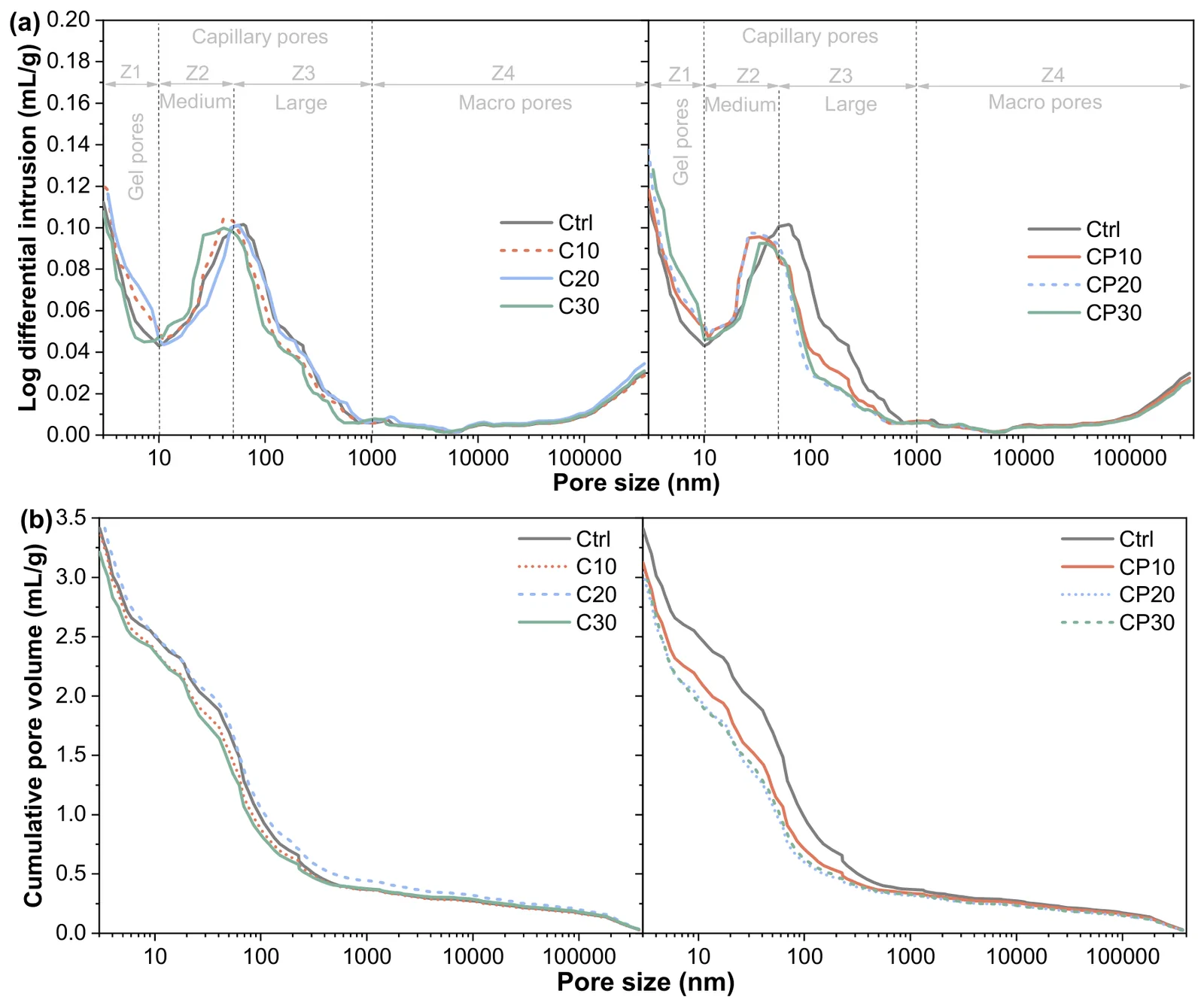
Pore structure parameters of the mixtures determined by MIP: (**a**) pore size distribution; (**b**) cumulative pore volume.

The pore characteristics of the hardened pastes containing dry and pre-wetted CGP were investigated by MIP, and the corresponding pore size distributions are presented in Figure 13. Based on the commonly accepted pore classification criteria [52,53], the pores were divided into four categories according to their characteristic sizes: gel pores (<10 nm), medium capillary pores (10–50 nm), large capillary pores (50–1000 nm), and macropores (>1000 nm). Among these pore types, capillary pores and macropores are closely associated with moisture transport, mechanical degradation, and durability deterioration, whereas gel pores generally contribute to matrix densification. As shown in Figure 13a, all mixtures exhibit two dominant pore populations located in the gel pore and capillary pore regions. The incorporation of CGP, regardless of its moisture condition, promotes an increase in gel pore volume (Z1), indicating the formation of additional fine pores within the hardened matrix. However, distinct differences were observed in the capillary pore distribution. The addition of dry CGP increased the overall capillary pore volume, although part of the pore structure shifted toward smaller pore sizes. This indicates that dry CGP mainly modifies pore refinement through particle filling and nucleation effects but provides limited regulation of internal moisture conditions.

In contrast, pre-wetted CGP exhibited a more beneficial influence on capillary pore development. Although a slight increase in capillary porosity was observed at lower incorporation levels, further increasing the pre-wetted CGP content resulted in a gradual reduction in capillary pores, approaching or even exceeding the refinement level of the reference sample. This behavior suggests that the internally stored water in pre-wetted CGP promotes sustained hydration, facilitating the generation of additional hydration products that progressively fill capillary voids and optimize the pore network. Furthermore, dry CGP caused a slight increase in macropore content (Z4), and this effect became more apparent with increasing replacement ratio, which may be associated with insufficient internal moisture supply and incomplete hydration of the surrounding cement matrix. In comparison, pre-wetted CGP effectively suppressed the formation of macropores and reduced the proportion of harmful pores, as demonstrated in Figure 13b. The refined pore structure and decreased connectivity of large pores contribute to a denser hardened matrix, thereby providing a microstructural basis for the improved mechanical performance of CGP-modified pastes.

#### 3.6.2. Nanoindentation

**Figure 14:**
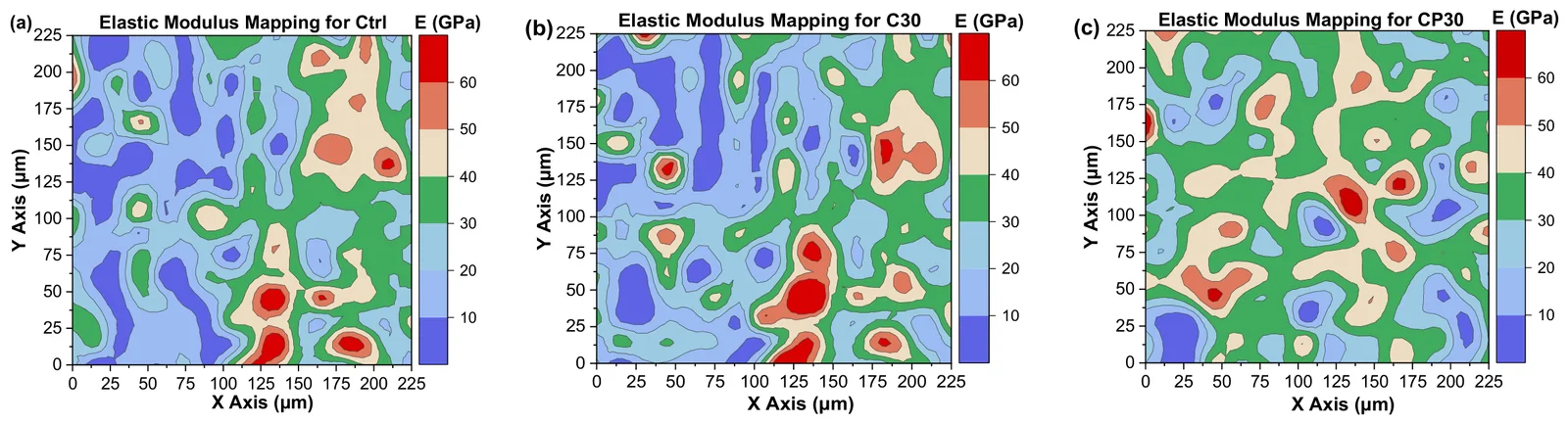
Two-dimensional contour maps of elastic modulus for the samples: (**a**) Ctrl; (**b**) C30; (**c**) CP30.

Nanoindentation was employed to assess the micromechanical properties of hardened pastes modified with dry and pre-wetted CGP after 28 days of curing. Elastic modulus mapping, presented in Figure 14, identifies regions above 50 GPa as unhydrated cement particles, while values below 50 GPa correspond to various hydration products [54]. The increased volume fraction of areas with elastic moduli in the 30–50 GPa range, attributed to CGP incorporation, reflects enhanced hydration and a greater abundance of hydration products. Pre-wetted CGP exhibits a more pronounced effect, leading to a substantial reduction in unhydrated cement content and demonstrating superior hydration promotion. However, both mixtures show an increase in low-modulus regions (<10 GPa), typically associated with pores or defective zones [55], suggesting that while CGP enhances hydration, it may also introduce microstructural heterogeneities that could partially offset mechanical improvements.

**Figure 15:**
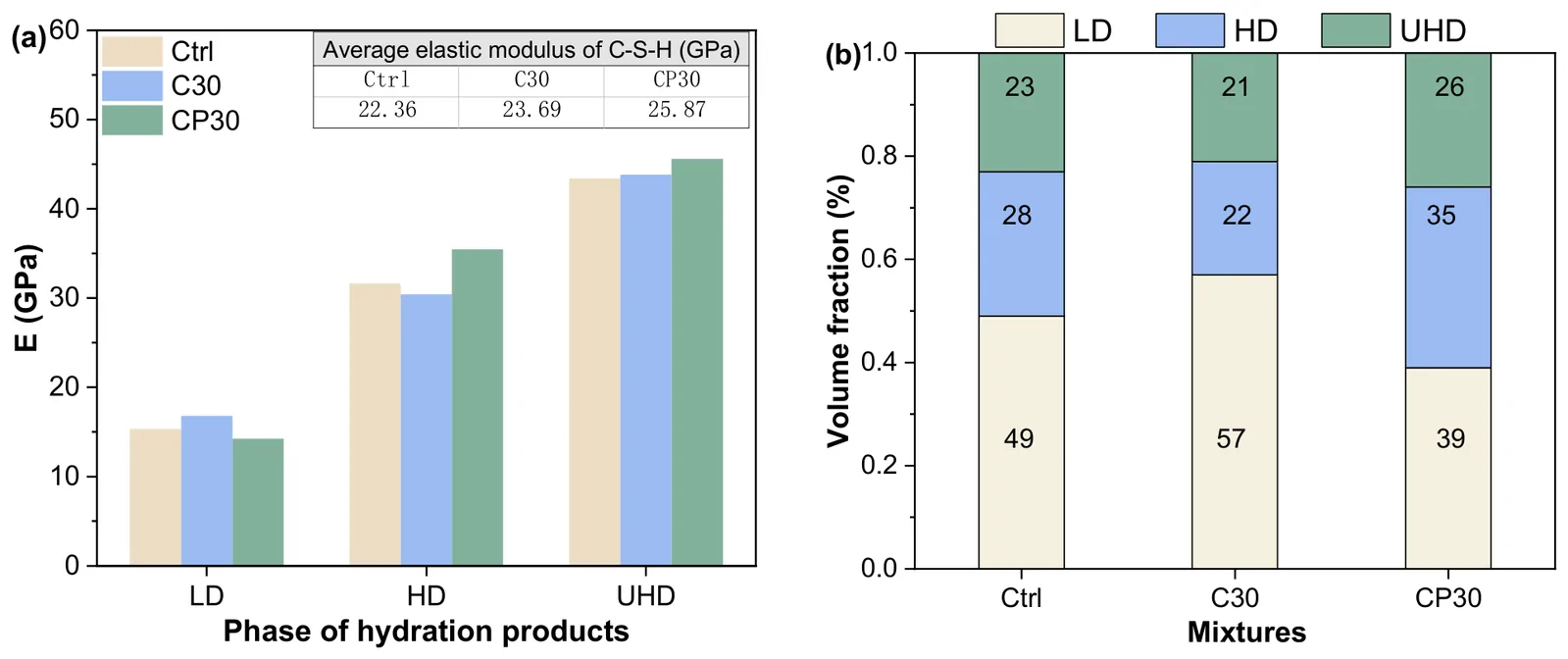
Deconvoluted elastic modulus distributions of the investigated samples. (**a**) conventional mixtures; (**b**) pre-wetted mixtures.

The elastic modulus distributions obtained from nanoindentation measurements were further analyzed using Gaussian fitting, as displayed in Figure 15a,b. Four characteristic regions were resolved from the fitted curves, corresponding to porous phases (PP), low-density calcium silicate hydrate (LD C-S-H), high-density calcium silicate hydrate (HD C-S-H), and ultra-high-density calcium silicate hydrate (UHD C-S-H), respectively [56]. In the fitting results, σ represents the distribution width, μ denotes the average elastic modulus of each constituent, and π indicates its relative volume fraction. Compared with the reference paste, the incorporation of dry CGP (C30) resulted in an increased proportion of LD C-S-H, rising from 49% to 57%, accompanied by a slight increase in its corresponding modulus. However, the contents and mechanical characteristics of HD C-S-H and UHD C-S-H remained almost unchanged. Consequently, the overall elastic modulus of the C-S-H phase exhibited no obvious improvement. These results indicate that dry CGP mainly affects the formation of relatively loose hydration products, likely through particle filling and nucleation effects, while its limited water supply restricts the further development of highly compact hydration structures.

A distinct modification of the hydration product distribution was observed in the paste containing pre-wetted CGP (CP30). The volume fraction of LD C-S-H decreased substantially to 39%, whereas the proportions of HD C-S-H and UHD C-S-H increased simultaneously, together with higher elastic modulus values. This transition from low-density to high-density C-S-H structures enhanced the average modulus of the composite matrix from 22.36 GPa for the control sample to 25.87 GPa. The improved micromechanical performance can be attributed to the internal curing effect provided by pre-wetted CGP, where the gradual release of stored water sustains hydration reactions and promotes the progressive transformation toward denser C-S-H structures. Therefore, pre-wetted CGP effectively optimizes the hydration product assembly and improves the mechanical integrity of the cementitious matrix at the microscale.

### 3.7. Comparison of Dry and Pre-Wetted CGP

Table 3 provides an integrated comparison of the performance of cement–fly ash composite matrices containing dry and pre-wetted CGP. The results show that CGP incorporation in the dry state mainly provides physical filling and nucleation effects, resulting in relatively limited improvements in hydration and shrinkage control. In contrast, pre-wetted CGP produces substantially greater effects across multiple properties. The pre-wetted CGP maintains a higher internal relative humidity and provides a sustained internal water supply, which promotes continued hydration and increases the main hydration peak. As a result, chemical, drying, and autogenous shrinkage are markedly reduced, while pore structure refinement and compressive strength development are enhanced. These effects become more pronounced with increasing CGP content, particularly for the CP30 mixture.

Overall, the comparison demonstrates that the beneficial effects of pre-wetted CGP cannot be attributed solely to its physical incorporation into the cement–fly ash matrix. Instead, its porous structure enables CGP to function as an internal water reservoir, which alleviates self-desiccation and sustains hydration during the early and later stages of curing. The resulting improvement in hydration and internal moisture conditions contributes to pore refinement, shrinkage mitigation, and mechanical performance development. Therefore, the key role of pre-wetting is to transform CGP from a predominantly passive mineral component into an effective internal-curing medium within the cement–fly ash composite matrix.

### 3.8. Discussion

#### 3.8.1. Relationship Between Shrinkage Behavior and Surface Tension

**Figure 16:**
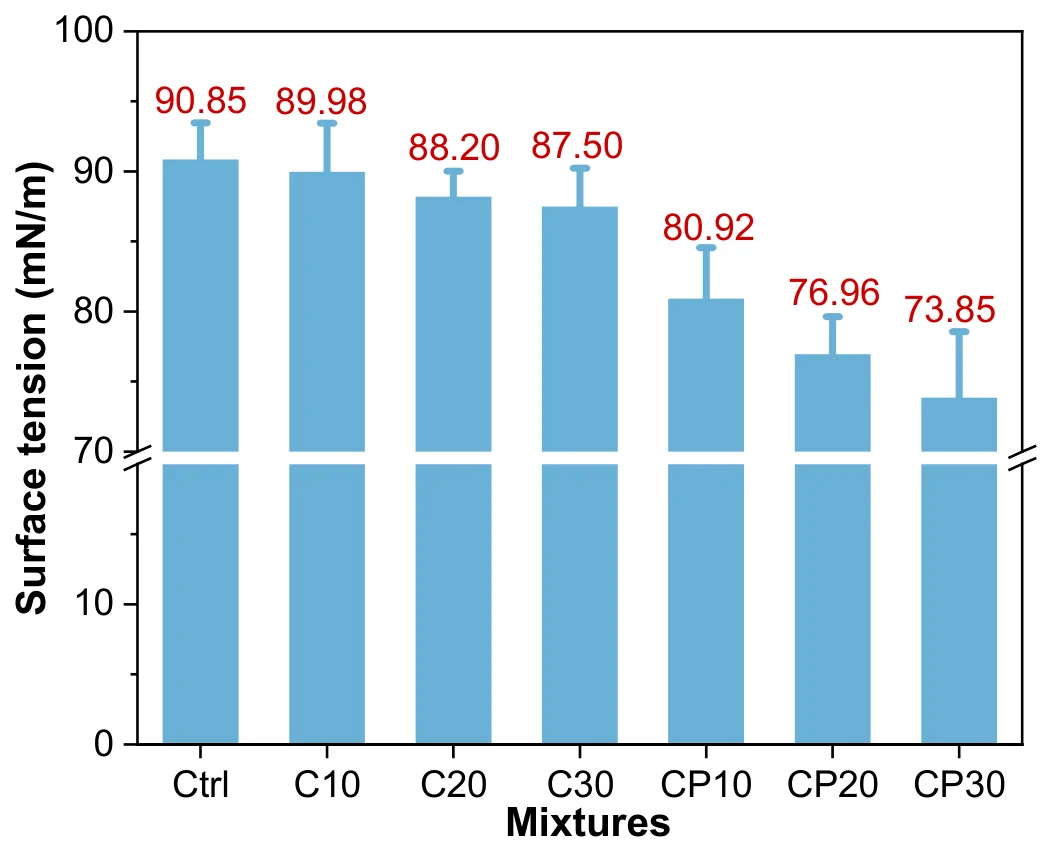
Measured surface tension values of the simulated pore solutions.

According to Laplace’s law [57], capillary pressure within the pore network of cement paste is governed by the surface tension of the pore solution, playing a critical role in autogenous shrinkage development. The surface tension of the pore solution directly influences the magnitude of capillary stress exerted on the solid skeleton; a higher surface tension leads to greater capillary pressure, which in turn promotes more pronounced autogenous shrinkage. The surface tension of the simulated pore solution decreased with CGP incorporation, as presented in Figure 16. Relative to the control, the reductions reached 1.0%, 2.9%, and 3.7% for mixtures C10, C20, and C30, respectively. This decrease implies that CGP contributes to lower capillary stresses and facilitates the stabilization of entrained air bubbles, thereby alleviating shrinkage-induced stress. The reduction in surface tension observed with dry CGP incorporation can be attributed to the adsorption of ions or fine particles at the liquid-vapor interface, which alters the interfacial energy of the pore solution.

Using pre-wetted CGP further enhances this reduction, yielding decreases of 1.6% (CP10), 3.7% (CP20), and 5.2% (CP30). This more pronounced lowering of surface tension significantly inhibits autogenous shrinkage, consistent with the results discussed in Section 3.8. The additional decrease in surface tension provided by pre-wetted CGP is likely associated with the gradual release of internal curing water, which may carry dissolved ions that further modify the pore solution chemistry and reduce interfacial tension. A significant reduction in autogenous shrinkage and enhanced dimensional stability is achieved by incorporating CGP, especially the prewetted form. This is accomplished through a dual effect: diminishing capillary pressure via reduced surface tension and promoting the stabilization of air bubbles within the paste. Consequently, the combined action of lower surface tension and improved internal moisture distribution synergistically contributes to the superior shrinkage resistance observed in pre-wetted CGP mixtures, particularly at higher replacement levels.

#### 3.8.2. The Role of Capillary Pore Pressure in Shrinkage

**Figure 17:**
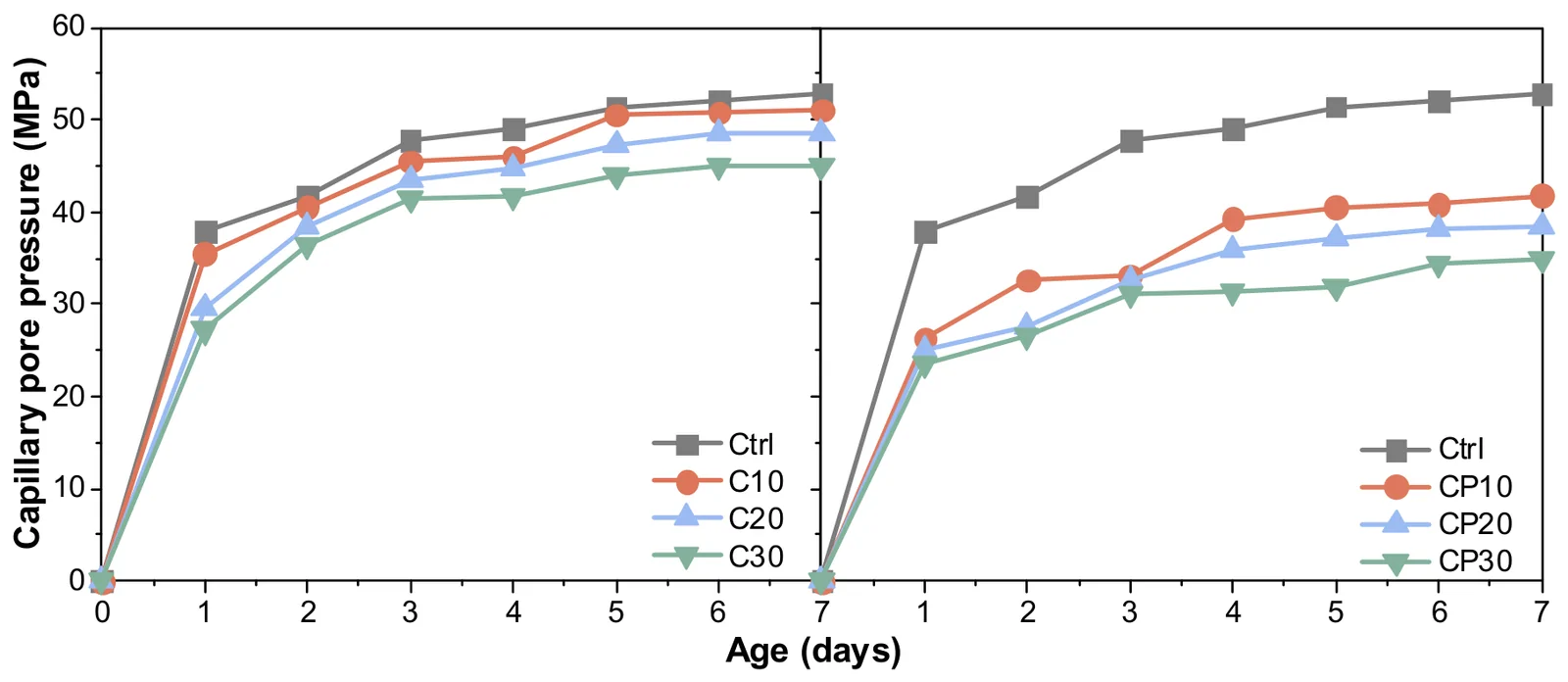
Development of capillary pore pressure for all mixtures.

Figure 17 presents the temporal development of capillary pore pressure within the cement paste systems. After initial setting, all formulations show a progressive rise in capillary pressure, culminating in a value of 52.6 MPa for the control mixture at 7 days. A low dosage of CGP exerts minimal influence on this trend. However, a 30% replacement with dry CGP (C30) reduces the 7-day capillary pressure by 14.6% compared to the control. This mitigation is associated with the hydration-enhancing role of CGP, where the consumption of capillary water and the consequent formation of hydration products lead to pore refinement, alleviating internal stress. A more pronounced and earlier reduction in capillary pressure is achieved using prewetted CGP, observable shortly after setting. The extent of pressure decrease is dependent on the replacement level, with the CP30 mixture exhibiting a 33.7% reduction relative to the control at 7 days. This superior performance stems from the internal curing capability of prewetted CGP. By supplying moisture to mitigate self-desiccation, it facilitates sustained hydration and fosters a more homogeneous microstructure. These combined actions effectively limit the buildup of capillary pressure and promote the formation of a denser, more dimensionally stable matrix.

**Figure 18:**
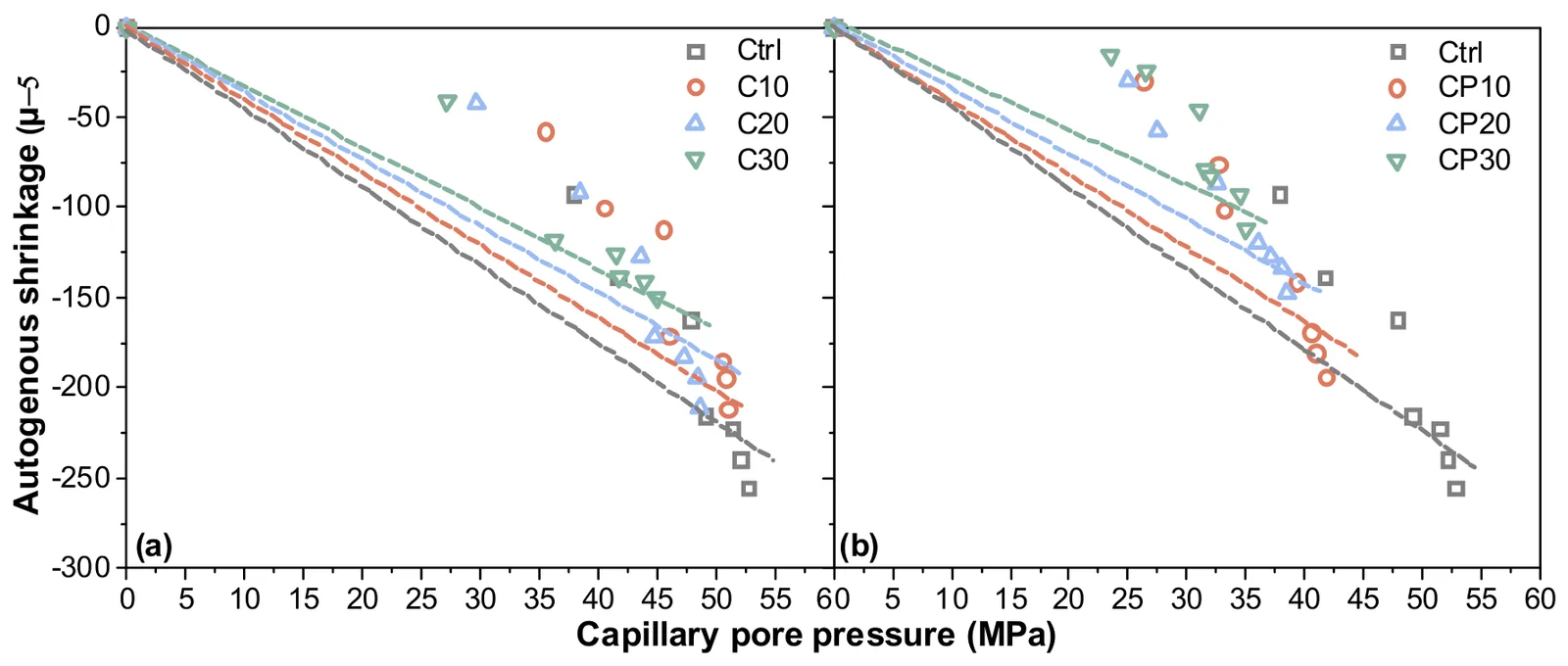
Relationship between capillary pore pressure development and autogenous shrinkage for the mixtures. (**a**) conventional mixtures; (**b**) pre-wetted mixtures.

The correlation between capillary pore pressure development and autogenous shrinkage in CGP-modified pastes, subjected to varying treatment methods, is analyzed in Figure 18. Results indicate that the sensitivity of shrinkage to capillary pressure varies significantly depending on the CGP processing approach. With increasing CGP replacement levels, the slope of the shrinkage–pressure relationship becomes progressively steeper. A key finding is that under equivalent autogenous shrinkage, mixtures with elevated CGP content correspond to consistently lower capillary pressure than those with lower dosages, with both cases remaining below the control level. This behavior implies that dry CGP affects autogenous shrinkage through mechanisms that extend beyond simple capillary effects, potentially involving microstructural modifications and changes in hydration progression. The introduction of prewetted CGP substantially alters this relationship. Under identical autogenous shrinkage conditions, mixtures containing higher proportions of prewetted CGP (e.g., CP30) display greater capillary pressure than those with lower content (e.g., CP10). This reversal suggests that prewetted CGP promotes more homogeneous water distribution within the paste, leading to improved hydration completeness. The internal water reservoir characteristic of prewetted CGP compensates for moisture deficits that typically occur with dry CGP, resulting in elevated capillary pressure at higher replacement ratios and consequently enhancing hydration efficiency. These results demonstrate that prewetted CGP enables synergistic management of autogenous shrinkage and capillary pressure, a key factor behind the performance gain in pastes, especially at higher CGP content.

A well-established fact is that the shrinkage behavior of cement paste is controlled by hydration kinetics, capillary pressure, as well as the properties of the internal pore system, particularly capillary pores ranging from 10 to 50 nm [58,59]. Pores below 50 nm exert considerable influence on drying shrinkage due to their role in amplifying capillary tension and promoting self-desiccation, mechanisms that are directly linked to autogenous shrinkage development [60]. MIP results presented in Table 4 provide quantitative insight into how CGP incorporation affects the pore structure within this critical size range (Z2 region). The data indicate a consistent reduction in both the number and volume of Z2 pores with increasing CGP content. For example, C30 exhibits a substantial decrease in pore volume compared to the control, implying that CGP particles either physically block or contribute to the refinement of these capillary pores, consequently modifying shrinkage performance. This pore-refining effect is further enhanced when prewetted CGP is used. With higher replacement levels of prewetted CGP, the reduction in Z2 pore volume becomes more pronounced, indicating significant microstructural improvement that contributes to superior shrinkage resistance.

### 3.9. Proposed Mechanism of Pre-Wetted CGP in Cement–Fly Ash Composites

**Figure 19:**
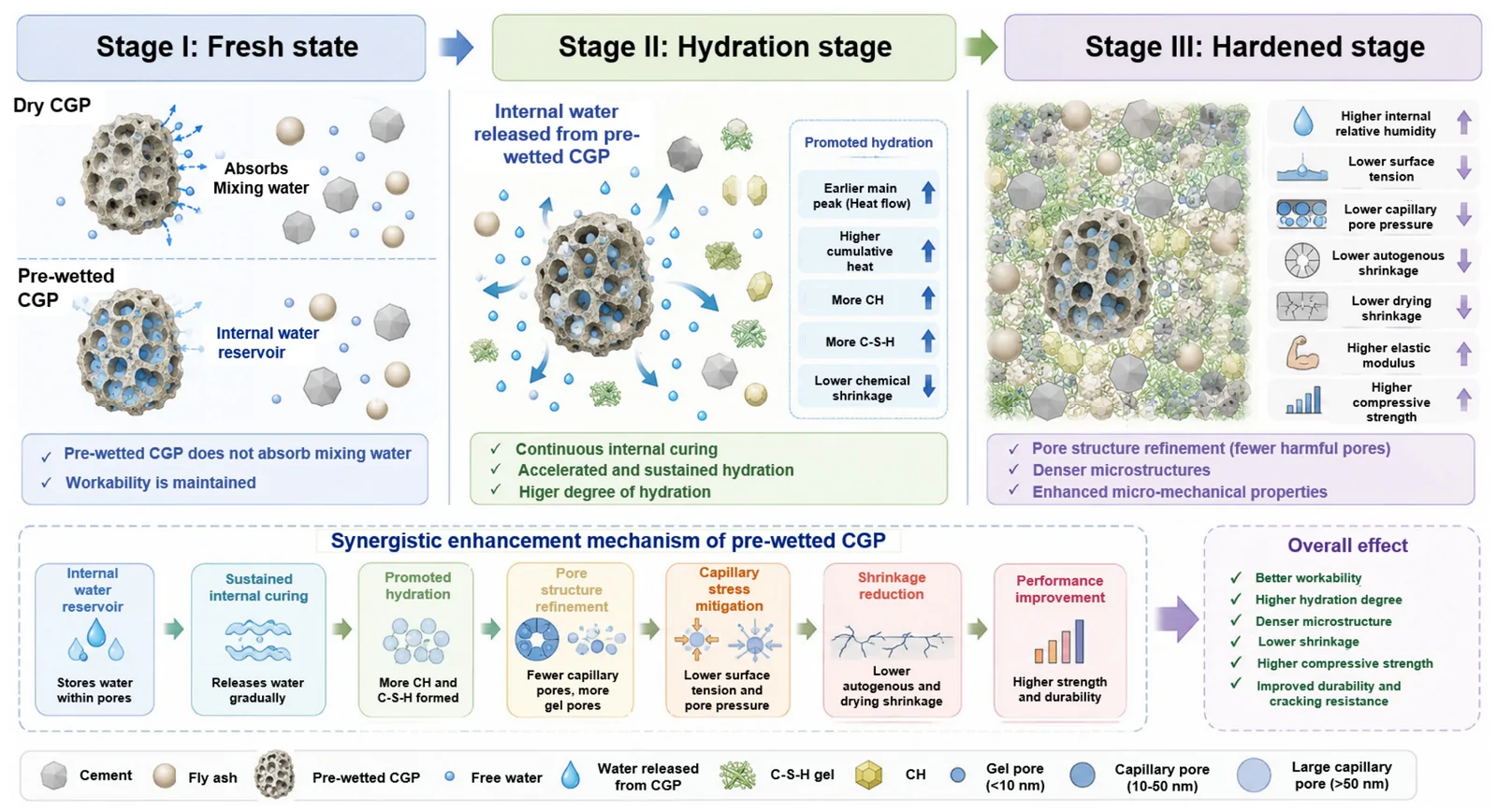
Schematic illustration of the synergistic enhancement mechanism of pre-wetted CGP in cement–fly ash composites.

The experimental results collectively demonstrate that the performance enhancement provided by pre-wetted CGP cannot be attributed to a single mechanism, but rather to the synergistic interaction among internal curing, hydration promotion, pore structure refinement, capillary stress regulation, and microstructural strengthening. These coupled mechanisms operate sequentially throughout the hydration process, ultimately leading to simultaneous improvements in workability, shrinkage resistance, and mechanical performance. Rather than acting independently, each process continuously influences the subsequent hydration evolution, resulting in a self-reinforcing enhancement pathway that links microscopic hydration behavior with macroscopic engineering performance. As schematically illustrated in Figure 19, the overall enhancement mechanism originates from the synergistic interaction among internal curing, hydration promotion, pore refinement, capillary stress regulation, and microstructural strengthening. To better illustrate these coupled interactions, Figure 19 schematically summarizes the evolution of the internal curing process, hydration promotion, pore structure refinement, capillary stress regulation, and microstructural strengthening induced by pre-wetted CGP, together with their relationships to the overall performance improvements observed in this study.

The enhancement process begins immediately after mixing. Unlike dry CGP, which rapidly absorbs mixing water and locally reduces the effective water-to-binder ratio, pre-wetted CGP already contains saturated water within its interconnected pore structure. Consequently, the particles function as distributed internal water reservoirs instead of water consumers. During the early hydration stage, the internal water stored inside the pre-wetted CGP is gradually released under the capillary pressure gradient generated by cement hydration. This continuous moisture supply compensates for the rapid consumption of free water caused by cement hydration, effectively alleviating self-desiccation and maintaining a more stable internal moisture environment [61,62]. Therefore, the detrimental reduction in workability commonly observed in mixtures containing dry CGP is effectively eliminated, while the hydration process proceeds under a more favorable water availability condition. As illustrated in Stage I of Figure 19, pre-wetted CGP changes its role from a water-absorbing particle to a distributed internal water reservoir, thereby providing the fundamental prerequisite for the subsequent hydration enhancement.

The sustained internal water supply subsequently promotes hydration reactions throughout the binder system. The calorimetry results demonstrated that pre-wetted CGP significantly accelerated the occurrence of the main hydration peak while increasing the cumulative heat release, indicating a higher hydration degree. Meanwhile, XRD and TG analyses confirmed the formation of larger amounts of hydration products, particularly portlandite and C–S–H gel. Unlike dry CGP, whose contribution is mainly limited to the physical filling effect and heterogeneous nucleation, pre-wetted CGP continuously supplies additional water during hydration, allowing previously unhydrated cement particles to participate in further reactions. This prolonged hydration process increases the production of hydration products over time, resulting in a more complete hydration process and a more homogeneous distribution of hydrates throughout the cement matrix. Therefore, the beneficial effect of pre-wetting extends beyond the acceleration of early hydration and continues to influence the later hydration stage through sustained internal curing. The continuous generation of hydration products further governs the evolution of the pore structure. As hydration proceeds, newly formed C–S–H gel progressively fills capillary pores and bridges adjacent solid particles, thereby refining the internal pore network [63,64]. The MIP results confirmed that pre-wetted CGP significantly reduced the proportion of harmful capillary pores while increasing the volume fraction of harmless gel pores. Simultaneously, nanoindentation analysis demonstrated a clear transformation from low-density C–S–H toward high-density and ultra-high-density C–S–H phases, accompanied by an increase in the average elastic modulus. These observations indicate that the additional hydration induced by internal curing not only increases the quantity of hydration products but also improves their spatial distribution and packing density. Consequently, the hardened matrix becomes denser, more continuous, and mechanically stronger than that obtained using dry CGP.

The refinement of the pore structure also fundamentally changes the moisture transport behavior within the cement matrix. As the capillary pore network becomes less connected and the internal relative humidity remains higher, the rate of self-desiccation is effectively suppressed. The higher IRH observed in mixtures containing pre-wetted CGP confirms that the internal water released from the saturated particles continuously compensates for moisture consumed during hydration. Meanwhile, the measured reductions in surface tension and capillary pore pressure indicate that the internal tensile stress generated within the pore solution is significantly alleviated. Since autogenous shrinkage is primarily driven by capillary pressure developed during self-desiccation, lowering both the surface tension and capillary pressure directly reduces the shrinkage-driving force [65,66]. Furthermore, the refined pore structure decreases the connectivity of large capillary pores, thereby limiting moisture evaporation during external drying. As a result, both autogenous shrinkage and drying shrinkage are substantially reduced through the combined regulation of moisture distribution, pore refinement, and capillary stress evolution. This coupled evolution is summarized in Stages II and III of Figure 19, where sustained internal curing promotes hydration, pore refinement reduces moisture transport pathways, and capillary stress regulation collectively contributes to the formation of a dimensionally stable cementitious matrix.

More importantly, these mechanisms are not independent but mutually reinforcing throughout the hydration process. The internal curing effect promotes hydration, while the increased hydration products continuously refine the pore structure. The refined pore structure, in turn, improves water retention capability, further sustaining hydration and maintaining internal relative humidity. Meanwhile, the higher hydration degree produces a denser C–S–H network with greater elastic modulus, which enhances the ability of the hardened matrix to resist deformation induced by capillary stress. Therefore, hydration promotion, pore refinement, capillary stress reduction, and microstructural strengthening together establish a positive feedback mechanism that continuously improves the overall performance of the cement–fly ash composite system. Consequently, the macroscopic performance improvements observed in this study, including enhanced fluidity, accelerated hydration, increased compressive strength, reduced chemical shrinkage, lower autogenous shrinkage, and improved drying shrinkage resistance, are all direct manifestations of this synergistic mechanism. Compared with conventional dry CGP, pre-wetted CGP fundamentally changes the role of CGP within the cementitious system, transforming it from a passive mineral filler into an active internal curing carrier capable of continuously regulating hydration kinetics, moisture transport, pore evolution, and stress development. Therefore, the superior performance of pre-wetted CGP originates from the cooperative interaction among internal water release, hydration enhancement, pore structure optimization, capillary stress mitigation, and microstructural densification, rather than from any individual mechanism acting alone.

## 4. Conclusions

This study systematically investigated the effects of dry and pre-wetted CGP on the hydration, moisture evolution, shrinkage, mechanical properties, and microstructural development of cement–fly ash composite pastes. Based on the integrated comparison, the main conclusions are as follows:

(1) Pre-wetting effectively regulates the fresh-state behavior of CGP-containing pastes. Compared with dry CGP, which strongly absorbs mixing water and consequently shortens setting time and reduces fluidity, pre-wetted CGP maintains a more favorable water distribution within the fresh matrix. At 30% CGP replacement, the fluidity of CP30 reached 185 mm, close to the 192 mm of the control and substantially higher than the 158 mm of C30.
(2) Pre-wetted CGP promotes hydration by providing a sustained internal water supply. At 30% replacement, CP30 increased the main hydration peak by 18.6% and advanced its occurrence by 1.2 h relative to the control, whereas the corresponding dry-CGP mixture showed a smaller increase of 8.8%. This indicates that the pre-wetting treatment changes CGP from a predominantly passive porous component into an effective internal-curing medium that sustains hydration.
(3) The internal-curing effect substantially mitigates shrinkage and improves moisture regulation. Compared with the control, CP30 reduced chemical, autogenous, and drying shrinkage by 36.5%, 47.4%, and 36.4%, respectively. The 7-day internal relative humidity reached 90.1%, while capillary pore pressure and pore-solution surface tension were reduced by 33.7% and 5.2%, respectively. These results demonstrate that sustained internal water release alleviates self-desiccation and reduces the capillary stress responsible for shrinkage.
(4) Pre-wetted CGP simultaneously improves pore structure and mechanical performance. CP30 increased the 28-day compressive strength by 6.2% compared with the control. MIP results showed a refined pore structure with reduced macropore volume, while nanoindentation indicated a shift toward denser C–S–H phases, with the overall elastic modulus increasing from 22.36 to 25.87 GPa. The coupled effects of enhanced hydration and microstructural densification contribute to the improved mechanical performance of the composite matrix.
(5) The comparative analysis demonstrates that pre-wetting is the key factor governing the enhanced performance of CGP in the cement–fly ash matrix. Dry CGP mainly contributes through physical filling and nucleation, whereas pre-wetted CGP additionally provides internal curing through controlled water release. The resulting coupling of sustained hydration, moisture regulation, pore refinement, and capillary stress reduction enables simultaneous improvement in shrinkage resistance and mechanical performance. Therefore, pre-wetting provides a simple and effective strategy for improving the utilization efficiency of mechanically activated CGP in cement–fly ash composite binders.
(6) The present findings provide laboratory-scale evidence for the potential application of pre-wetted CGP in sustainable cementitious materials. However, long-term durability, contaminant leaching and immobilization, process-scale energy consumption, economic feasibility, and life-cycle environmental impacts were not quantitatively evaluated in this study. Further research is therefore required before large-scale engineering implementation.

## Figures and Tables

**Table 1:** Chemical constituents of the raw materials (wt.%).

	Fe_2_O_3_	CaO	SiO_2_	MgO	TiO_2_	Na_2_O	Al_2_O_3_	K_2_O	Other
Cement	0.90	44.69	25.49	3.89	1.11	0.11	19.21	0.81	3.79
CGP	9.72	1.55	57.25	0.47	0.55	0.78	26.49	0.35	2.84
Fly ash	3.21	2.51	58.01	0.71	2.79	0.07	25.09	1.29	6.32

**Table 2:** Mix proportions/(g/100 g).

Mixtures	Cement	Fly Ash	CGP	*w*/*b*
Ctrl	80	20	0	0.33
C10	72	18	10	0.33
C20	64	16	20	0.33
C30	56	14	30	0.33
CP10	72	18	10	0.33
CP20	64	16	20	0.33
CP30	56	14	30	0.33

**Table 3:** Integrative comparison of dry and pre-wetted CGP in cement–fly ash composite matrices.

Performance	Dry CGP	Pre-Wetted CGP	Effect of Internal Curing
Hydration	Main peak slightly enhanced; C30: +8.8%	Main peak markedly enhanced; CP30: +18.6%	Sustained internal water availability promotes hydration
Chemical shrinkage	Reduction: 1.7–8.6%	Reduction: 20.0–36.5%	Reduced self-desiccation
Internal RH	Limited improvement	CP30: 90.1% at 7 d	Maintains a higher internal moisture state
Drying shrinkage	—	Reduction: 20.6–36.4%	Internal water release mitigates moisture loss
Autogenous shrinkage	Reduction: 3.9–15.7%	Reduction: 19.3–47.4%	Stronger suppression of self-desiccation
Compressive strength	Limited improvement	CP30: +11.7%, +5.5%, +6.2% at 3, 7, 28 d	Promotes sustained hydration and strength development
Pore structure	Increased capillary porosity	Finer pore structure	Pore refinement through continued hydration
Overall effect	Mainly physical filling/nucleation	Internal water reservoir	Pre-wetting transforms CGP into an effective internal-curing medium

**Table 4:** Cumulative pore volume of different pore zones (Z1–Z4)/(mL/g).

Pore	Ctrl	C10	C20	C30	CP10	CP20	CP30
Z1	0.878	0.932	0.880	1.033	0.919	0.938	1.005
Z2	0.952	0.926	0.837	0.879	0.864	0.863	0.840
Z3	0.349	0.475	0.610	0.532	0.350	0.465	0.517
Z4	0.385	0.333	0.339	0.352	0.375	0.278	0.330

## Data Availability

The original contributions presented in this study are included in the article. Further inquiries can be directed to the corresponding author.
